# ORAT: Detector-Side Identity and Observation Relations for Online Multi-Pig Tracking

**DOI:** 10.3390/s26216691

**Published:** 2026-10-22

**Authors:** Sejun Lee, Kijeong Nam, Seungwook Son, Jonggwan Kim, Shinhoo Kang, Yongwha Chung

**Affiliations:** 1 Info Valley Korea Co., Ltd., Anyang 14067, Republic of Korea; lllss22@invako.kr (S.L.); nam9072@invako.kr (K.N.); sso7199@invako.kr (S.S.); jking@invako.kr (J.K.); 2 Department of Computer Software, Korea University, Sejong 30019, Republic of Korea

**Keywords:** multi-object tracking, precision livestock farming, detector-side identity, observation relations, online association, edge inference

## Abstract

**Highlights:**

**What are the main findings?**

- Adding the recent-memory configuration, which retains individual recent observations, increased mean validation Higher Order Tracking Accuracy (HOTA) across 5 seeds on PigTrack and SportsMOT. In local comparisons using identical D-FINE-X detections on PigTrack, the base Observation- and Relation-based Affinity Tracker (ORAT) configuration achieved higher HOTA, Association Accuracy (AssA), and Identification F1 Score (IDF1) than the comparison trackers.
- On both the server and Jetson, ORAT achieved higher throughput than comparison methods using external re-identification (ReID), and higher HOTA than ByteTrack, OC-SORT, and FTO-SORT.

**What are the implications of the main findings?**

- Evaluations on two pig datasets and two human tracking datasets, with separate training on each human dataset, showed that ORAT’s association architecture, designed primarily for pigs, can also be applied to human tracking with similar appearances and complex motion.
- Avoiding additional ReID inference can be useful in settings that require consideration of both tracking accuracy and throughput.

**Abstract:**

Similar appearances, frequent contact, and occlusion make current and past appearance cues necessary for reliable pig association. External re-identification (ReID) requires an additional forward pass for appearance extraction, while the extent and use of stored appearance history affect accuracy and computational cost. We propose ORAT (Observation- and Relation-based Affinity Tracker) for online pig tracking. Bounding boxes and identity embeddings are generated from the same queries in one detector forward pass, avoiding separate cropping and ReID inference. Each track maintains cumulative state and bounded memory of accepted recent observations. Relations between current detections and stored observations are recomputed each frame using appearance similarity and elapsed time. With identical D-FINE-X detections on the PigTrack test, base ORAT achieved a Higher Order Tracking Accuracy (HOTA) of 88.1 and server full-pipeline throughput of 48.6 frames per second (FPS). HOTA jointly evaluates detection and association. On private Jochiwon23 videos, ORAT outperformed DiffMOT in association metrics under outer-test evaluation separated from internal model selection. We applied the same architecture to human tracking by training separately on SportsMOT and DanceTrack. In component experiments across five training seeds, adding recent memory increased mean validation HOTA on PigTrack and SportsMOT.

## 1. Introduction

For group-housed pigs, group-level observations or information from a single time point are often insufficient to identify individual health conditions and abnormal behavior promptly. Continuous observation at the individual level is therefore needed to detect changes in health, welfare, and production status at an early stage [1,2]. To meet this need, artificial intelligence (AI)-based precision livestock farming technologies are being investigated to analyze individual activity levels, movement trajectories, and social contacts over time [3]. Although animal-mounted sensors can also be used for condition monitoring, video analysis with fixed cameras provides a noncontact monitoring approach: it allows the same housing area to be observed over extended periods without attaching devices to pigs [4].

Video analysis with fixed cameras is used to characterize pig posture, activity levels, social contacts, movement, and behavioral patterns [5,6,7,8]. These individual-level analyses begin with detection, which identifies the location and bounding box (bbox) of each pig in a frame. However, single-frame detection provides only observations from the current frame and does not indicate whether detections in different frames correspond to the same individual. Analyzing temporal changes in individual condition and behavior therefore requires linking observations across frames while preserving identity. Multi-object tracking (MOT) provides the foundation for constructing individual time series by linking detections of the same pig across frames into a single track [4].

Typical online tracking-by-detection methods for MOT update tracks by determining one-to-one associations between current-frame detections and existing tracks [9,10]. Maintaining correct associations over extended periods in pig-farm videos, however, requires addressing several challenges arising from the animals and their housing environment. Pigs of the same breed housed in a pen may have similar appearances, while irregular movement and nonrigid changes in posture further complicate association [4,11]. In densely populated pens, frequent contact and overlap, together with facilities and camera viewpoints, can cause partial or complete occlusion, potentially leading to identity (ID) switches or track fragmentation [6,12].

To improve association quality, offline trackers have been proposed that use observations from future frames or the entire sequence to link tracks across occlusion or correct them retrospectively [13]. Although access to future observations makes offline trackers useful for retrospective analysis of recorded videos, it limits their ability to provide current results continuously in input order. Online trackers instead update tracks using only current and past observations, without access to future frames [14]. Online MOT is therefore better suited to this study, which targets continuous individual monitoring and low-latency output.

Deploying an online tracker in a pig farm requires consideration of overall processing speed as well as association accuracy. If the full pipeline cannot keep pace with the incoming video, delays accumulate or frames must be skipped, limiting its usefulness for low-latency monitoring. On-site devices may need to perform both detection and tracking with limited computational resources [15,16]. Here, end-to-end (E2E) covers the complete pipeline from video input to final track output. We assess tracking accuracy together with throughput in frames per second (FPS) and latency per frame over this scope.

External re-identification (ReID), which identifies the same individual across detections in different frames, and observation memory are common approaches to improving association accuracy under these deployment requirements, but both incur additional costs. A separated tracker using external crop-based ReID crops each detection bbox and generates appearance features with a separate ReID model. These features help distinguish pigs with similar appearances and preserve identity after temporary occlusion and missed detections. However, bbox crop and an additional forward pass for each detection can reduce full-pipeline throughput [10]. Observation memory preserves temporal changes in motion and appearance, but the benefit of using history depends on the temporal interval [17]. Contamination and aging of accumulated appearance information must also be considered [18], and stale features in an excessively long history can reduce performance [19]. Because the number of retained observations also affects storage requirements and the cost of computing relations, we limit the memory capacity accessed by each candidate pair (Section 3.3). Thus, beyond history length, it is important to consider how individual appearance observations and their timestamps, retained in bounded memory, are used to decide whether to associate a track with the current detection. Online multi-pig tracking requires a design that reduces the cost of external appearance extraction while using stored observations as relevant evidence for associating the current detection.

To address these trade-offs, we propose the Observation- and Relation-based Affinity Tracker (ORAT), a learnable association tracker built on the D-FINE detector [20]. The method estimates affinity using both representations obtained from the current detection and observation information retained in the track, together with relations between the current detection and the track or its stored observations. This design enables ORAT to learn from data how to use multiple tracking cues to estimate affinity, rather than relying solely on predefined similarity criteria. ORAT also transforms the detector’s final query through a dedicated identity head to generate a box-aligned identity embedding corresponding to the bbox at the same query index. This preserves the correspondence between detections and identity representations while avoiding separate bbox cropping and an external ReID forward pass. The bbox, detection confidence, and localization quality obtained in the same detector forward pass also serve as association cues.

Each track retains a cumulative identity summary alongside bounded memory containing the identity embeddings and observation timestamps of accepted recent detections. The method computes similarities between the current detection and individual observations stored in the track, allowing stored observations that closely match the current detection to inform association. The maximum similarity and the mean similarity of the highest-ranked observations indicate the strength of past appearance evidence supporting that detection, while the age of the most similar observation indicates when that evidence was acquired. ORAT combines these relations with detector-side representations and cumulative track state to estimate detection–track affinity. This enables appearance changes associated with posture or occlusion to be compared against individual observations rather than relying on a single summary.

This study makes three contributions.

- **Recent-memory association using observation relations.** Individual recent observations are retained separately from the cumulative identity summary. Similarity statistics between the current detection and stored observations, together with observation timestamps, inform the learned affinity used for one-to-one assignment. In component experiments using five fixed training seeds on PigTrack and SportsMOT, adding the recent-memory configuration increased mean validation Higher Order Tracking Accuracy (HOTA) (Section 4.3.2 and Section 4.5.2).
- **Detector-side identity representations.** The detector’s final query is transformed into an identity embedding aligned with the corresponding bbox, providing the representation needed for association without separate cropping or an external ReID forward pass. On PigTrack, we compared D-FINE queries and dedicated head outputs with the detector, bboxes, and dimensionality (256) held constant, and also compared the 512-dimensional external OSNet and internal identity-embedding paths using the same detections [20,21]. With a tracker trained for each representation, the dedicated head path achieved higher HOTA and similar throughput compared with the pre-head query of the same dimension. The internal 512-dimensional path achieved higher HOTA and throughput than external OSNet (Section 4.3.1).
- **Multi-dataset evaluation on pigs and humans and accuracy–throughput assessment.** Tracking performance across different pig videos was evaluated using the test split of the public pig benchmark PigTrack and private Jochiwon23 videos collected at an operational pig farm [11]. Jochiwon23 was evaluated in two directions, with internal training and validation separated from outer testing, using videos from the same farm and herd. The same association architecture was also trained, selected, and evaluated separately on SportsMOT and DanceTrack, which share the challenges of similar appearances, irregular motion, and occlusion [22,23]. These evaluations show that the architecture designed primarily for pigs can also be applied to human tracking after dataset-specific training. To assess its practical value in terms of association performance and computational cost, we also analyzed full-pipeline accuracy and throughput within each device on a server and Jetson Orin Nano Super (NVIDIA Corporation, Santa Clara, CA, USA).

## 2. Related Work

In video-based pig monitoring, frame-level detections and temporally linked observations of individuals provide inputs to downstream tasks such as counting [15] and the analysis of movement, contact, and behavior [6,7,8]. EmbeddedPigCount combined pig detection and tracking on an embedded board and evaluated both counting performance and computational cost [15]. An audiovisual study combined pig regions detected in video with acoustic information to localize coughing individuals [24].

In pig farms monitored by fixed cameras, backgrounds and facilities, lighting, camera placement, and domain differences between farms can affect the quality of the frame-level detections supplied to a tracker. StaticPigDet introduced a fixed-camera pig detection method that uses background and facility information obtained from long-duration videos [25]. StaticPigDetv2 incorporated depth-based background and facility information to address detection robustness in unseen environments [26], while a self-training study proposed domain adaptation using limited target annotations [27]. These detection studies address the reliability of the input observations required for tracking. Distinct from these application and detection studies, the following sections position ORAT relative to prior work on identity representations within detectors, association across frames, and memory for past observations.

### 2.1. Pig Identity Representations and Detector-Side Integration

Pigs of the same breed often have similar appearances, while nonrigid changes in posture and orientation, contact, occlusion by facilities, and lighting changes recur in pig videos. As a result, current-frame position or a single appearance cue is often insufficient to distinguish identities [4]. Maintaining track identity over a short interval and identifying an individual over extended periods or after reappearance through ReID also involve different temporal scopes and evaluation conditions. Psota et al. addressed long-term individual identification of group-housed livestock using keypoint detection and maximum a posteriori (MAP) estimation [28]. PigTrack provides a dataset for systematically comparing pig MOT methods under diverse real-world pig-farm conditions, including occlusion and low visibility [11], while OinkTrack provides a dataset for jointly studying ultra-long-term pig tracking and ReID [12]. Pig tracking thus requires identity representations that can be compared over time, alongside positional cues.

External crop-based ReID extracts an individual region from each bbox produced by the detector and runs a separate appearance network to obtain an embedding [10]. This architecture allows the detector and appearance model to be replaced independently. However, cropping and additional forward passes increase with the number of detections per frame, making it necessary to distinguish tracker-only speed from full-pipeline processing time. In pig tracking, methods such as Improved DeepSORT have incorporated appearance cues obtained from a separate appearance network into association [29]. Subsequent studies combined DeepSORT-based pig ReID with spatial-state correction [30], or used an improved appearance network and complete intersection over union (CIoU) for association [31]. As an alternative to these external appearance paths, ORAT generates identity embeddings from internal detector representations without separate crop extraction or a ReID forward pass.

Methods that share detection and identity learning aim to learn the representations needed for association while reducing reliance on a separate external ReID network. Pig tracking studies have proposed combining detection and identity embeddings through joint detection and embedding (JDE) [32], addressing long-term pig ReID within a one-shot architecture [33], and using weighted association while comparing JDE, FairMOT, and DeepSORT [34]. JDE and FairMOT for general-purpose MOT likewise generate detection and identity cues from shared representations [35,36].

DETR-family detectors predict classes and bboxes from object queries, with a direct correspondence between each final query and its predicted bbox within a frame [20,37]. In pig tracking, STARFORMER applied spatiotemporal contrastive learning to object-level embeddings from a DETR decoder to learn identity-discriminative representations for tracking and long-term ReID [38]. FDTA analyzed the high inter-object similarity of object embeddings produced by a shared DETR and used spatial, temporal, and identity adapters to learn instance-discriminative embeddings [39]. ORAT preserves D-FINE’s within-frame query–bbox correspondence and uses a dedicated Query ID Head to generate an identity embedding that directly corresponds to each detection.

### 2.2. Online Data Association for Multi-Pig Tracking

Online tracking-by-detection constructs costs or affinities between tracks maintained through the previous frame and current detections, then performs one-to-one matching. SORT uses a Kalman filter and intersection over union (IoU), while DeepSORT adds a separate appearance metric [9,10]. ByteTrack incorporates low-score detections into staged association, whereas OC-SORT emphasizes motion updates based on observed bboxes [40,41]. Research on pig videos has expanded from individual detection and tracking in commercial farms [4] to adapting detectors, appearance cues, thresholds, and lifecycle rules to farm conditions.

Association research tailored to pig-farm environments addresses several distinct aspects: combining appearance and geometry, recovering from occlusion, maintaining reliability over sparse intervals, and managing track lifecycles. The PigsTrack tracker uses multi-stage association combining appearance, IoU, and occlusion-recovery matching [42]. FTO-SORT removed BoT-SORT’s external ReID, adjusted track thresholds, and added a Farm Track-ID Optimizer for unseen pig-farm environments [43]. STA-PigTrack uses appearance-independent spatiotemporal association [44], while SIPTrack addresses reliability-aware identity prediction over sparse intervals [45].

Nevertheless, the evaluation coverage of pig-farm MOT studies remains limited. Henrich et al. noted that existing public pig detection and tracking data have limited environmental diversity and are rarely reused for comparisons across studies [11]. The review by Nidhi et al. likewise reported that livestock MOT research remains limited and that recent MOT methods have primarily been evaluated on pedestrian and vehicle benchmarks [46]. The hidden Markov model (HMM)-based study by Ngo Bibinbe et al. provides an example of extending the evaluation of a pig tracking method to human videos. It combined intermittent identification information from a feeder with tracking results and evaluated its effects on pig videos and MOT17 and MOT20. In the MOT17 and MOT20 experiments, intermittent identification cues simulated from ground-truth identities were supplied as additional inputs during inference [47]. However, its offline retrospective correction framework uses both intermittent identification information and future observations, so its inputs and execution conditions differ from those of online association using only current and past video frames [47].

For general-purpose MOT, methods have been proposed that directly model temporal geometry or track history to learn online association. TWiX/C-TWiX learns affinities between tracks and detections from different temporal segments using only bbox coordinates [48], while TDLP predicts link probabilities between track histories and current detections [49]. DiffMOT extends motion cues by learning to predict nonlinear motion from past trajectories rather than learning association itself [50]. TBDQ-Net learns interactions between detection queries from a frozen detector and previous track queries through a lightweight associator [51]. ORAT adopts learned online association for multi-pig tracking using video-derived cues without additional identification devices. It retains the tracking-by-detection execution boundary in which a separate association stage processes current-frame detector outputs. It learns an affinity for each track–detection pair using identity, bbox geometry, and relations to a bounded set of observations. During inference, a fixed geometry gate restricts the matching candidates. In addition to testing on videos from the public PigTrack dataset and performing two-direction outer-test evaluation, separated from internal model selection, on separately collected private pig-farm videos, we evaluate the same association architecture separately on SportsMOT and DanceTrack [22,23]. These evaluations assess its applicability across videos featuring similar appearances and complex motion.

### 2.3. Temporal State and Memory for Long-Term Identity Continuity

Long-term identity continuity in pig tracking can be disrupted by partial or complete occlusion, consecutive missed detections, exits from and reentries into the field of view, sparse sampling, and appearance changes across recording times [12,33,45]. Under these conditions, it is important to distinguish between updating only the last bbox or the most recent appearance observation and reconnecting interrupted tracks using identity cues accumulated over extended periods. Psota et al. addressed long-term livestock identification using keypoints and MAP estimation [28], while OinkTrack provides a dataset and benchmark for evaluating identity continuity in ultra-long-term pig tracking and ReID [12]. SIPTrack also considers the reliability of identity prediction over irregular sparse intervals [45]. These studies show that long-term pig tracking requires identity state maintained over time, in addition to current observations.

Long-term temporal state can take different forms depending on which past information is retained. In pig tracking, a method using an identity memory bank and trajectory relinking has been proposed to reconnect interrupted trajectories using identity information retained over extended periods [52]. In general-purpose MOT, GHOST uses recent appearance for active tracks and an aggregate distance between stored appearance history and the current detection for inactive tracks [53]. HMP maintains both long-term appearance prototypes and a short first-in, first-out (FIFO) buffer of recent observations, using accumulated identity summaries alongside the latest observations [18]. These approaches show that past information can either be compressed into a prototype or aggregate representation, or retained as individual observations from different times. The former provides an identity representation that aggregates multiple observations, whereas the latter preserves information for directly comparing recent appearance changes and temporal relations with the current detection.

Learned memory methods have recently been proposed that integrate temporal history directly into the construction of association representations rather than using it through fixed distance rules. MeMOT and MeMOTR incorporate long-term memory into the generation and updating of track representations [54,55], while Gated Temporal Fusion Transformer adaptively combines multi-frame tracklet memory [56]. PuTR encodes a bounded sequence of recent observations with a Transformer to use temporal context for association [57]. HAT, by contrast, adapts external ReID features to the current sequence through a Fisher linear discriminant transformation based on accumulated history, without additional neural network training [19]. Instead of compressing the entire history into one aggregated track representation, ORAT maintains a cumulative identity summary and a bounded set of individual recent observations in parallel. This dual-state memory preserves both identity cues aggregated across multiple observations and information from individual recent observations, allowing different observation relations and temporal attributes to be constructed for the current detection candidate. However, ORAT’s memory applies only to tracks retained within the maximum missed-observation gap; it does not perform retrospective relinking of terminated trajectories or separate ReID for exits from and reentries into the field of view.

## 3. Materials and Methods

This section describes how ORAT associates detections with track states under online conditions. We first define the frame-by-frame processing flow and input sets, then describe the detector that generates box-aligned identity representations, the memory that retains past observations and their relations to current detections, and the architecture that learns affinities. Finally, we present the procedures for determining final matches through one-to-one assignment and threshold-based acceptance and for managing track lifecycles.

### 3.1. Online Problem Formulation and Processing Flow

ORAT is an online tracker that processes each frame once, in arrival order. It therefore does not apply backward smoothing using future observations, sequence-wide relinking, or offline trajectory merging, nor does it revise previously output matches using information from future frames. Each frame is processed through candidate generation, affinity computation, one-to-one assignment, threshold-based acceptance, state updates, and lifecycle management, in that order. The cumulative identity summary and observation memory are updated only from finally accepted correspondences, and the updated information is used for association in the next frame.

The set of valid detections used as tracker inputs at time *t* is defined as $D^{t}=\{d_{j}^{t}{\}}_{j=1}^{N_{D}}$. This set contains only detections that pass both the cache-generation threshold and the tracker-input threshold; the conditions for these two filtering stages are described in Section 4.1.3. The set of active tracks retained within the maximum missed-observation gap is denoted by $T^{t}=\{T_{i}^{t}{\}}_{i=1}^{N_{T}}$. Whether track *i* and detection *j* satisfy the fixed geometric candidate gate is indicated by $C_{\mathrm{ij}}^{t}\in \{0,1\}$. During inference, only pairs that pass this gate are considered as matching candidates. Affinity is computed by adding the scores from the representation and relation paths described in Section 3.4. The representation path forms track and detection representations using information from the current detector outputs and cumulative track states, then computes a representation-distance score from the distance between the two representations. The localization-quality descriptor is incorporated into this path. The relation path computes a Pair-Bias score from pairwise relations between the current detection and the track or its stored observations. The affinities of candidate pairs that pass the gate are used to obtain Hungarian one-to-one assignment proposals. Only proposals meeting the affinity threshold are accepted as final matches. The assignment and acceptance procedures are detailed in Section 3.5.

### 3.2. Query-Aligned Detector Outputs

**Figure 1:**
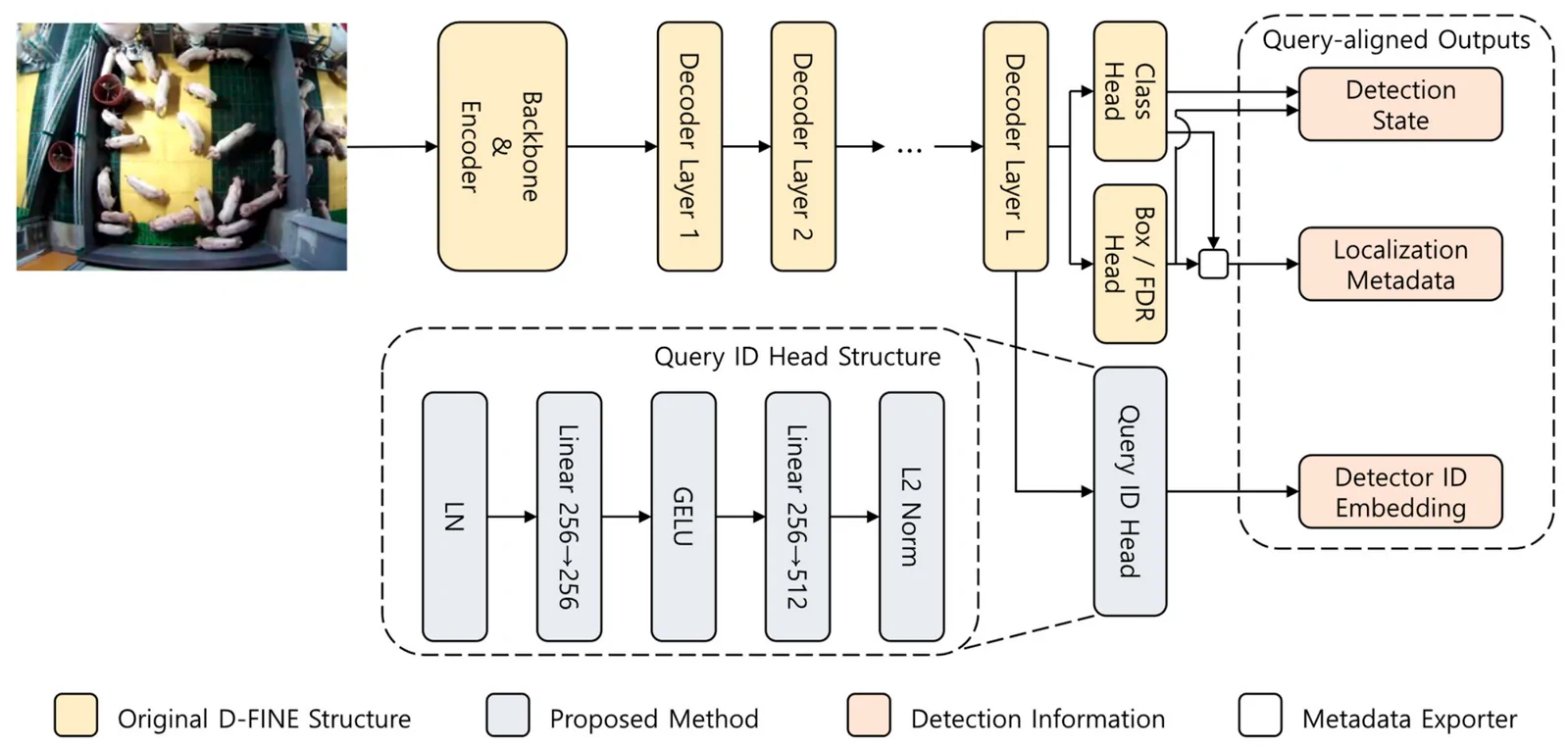
D-FINE detector extension for providing information needed for tracking. The Query ID Head, placed in parallel with the existing class and bbox heads, transforms a 256-dimensional query into a 512-dimensional L2-normalized identity embedding. The bbox, detection confidence, identity embedding, and 13-dimensional localization-quality descriptor are aligned by the same query index. Arrows indicate data flow; dashed outlines group outputs or show the expanded head structure. The ellipsis denotes intermediate decoder layers. Abbreviations: bbox, bounding box; ID, identity; FDR, fine-grained distribution refinement; LN, layer normalization; GELU, Gaussian error linear unit; L2 Norm, L2 normalization.

The first component of ORAT is a detector interface that outputs detections together with identity representations. D-FINE predicts classes and bboxes from object queries in the final decoder layer [20]. ORAT retains the existing class and bbox heads while adding a Query ID Head and a deterministic localization-quality path. The Query ID Head transforms each final 256-dimensional query into an identity embedding, while the localization-quality path computes a localization-quality descriptor. The bbox, detection confidence, identity embedding, and localization-quality descriptor obtained in one detector forward pass are aligned by the same query index. Figure 1 illustrates this process.

A detection query **q***_j_* ∈ ℝ^256^ is transformed into an identity representation by the dedicated Query ID Head. The head sequentially applies layer normalization (LN), a 256-dimensional hidden projection, Gaussian error linear unit (GELU), and an output projection, followed by L2 normalization.

**e***_j_* = norm_2_ (*W*_2_ GELU (*W*_1_LN(**q***_j_*) + **b**_1_) + **b**_2_),  **e***_j_* ∈ ℝ^512^ (1)

The identity branch operates in parallel with the class and bbox heads, and its embeddings are not fed back into the detection heads. During detector training, however, gradients from the identity loss propagate to the shared decoder.

**Training data for detector identity learning.** Identity supervision is applied only to regular object queries in the final decoder layer that the detector’s Hungarian matcher assigns to ground-truth bboxes during training. Background queries and invalid identity labels are excluded from supervision. Identity label spaces are separated by sequence to prevent collisions between identical integer IDs in different sequences.

Each mini-batch for the identity loss consists of frames selected from a single sequence, and positive and negative pairs are formed within that mini-batch. We do not use cross-sequence correspondence information to merge the same individual appearing in different sequences into one identity. Thus, the same pig may have different identity labels in different sequences.

Identity representations are learned using the cosine similarities of the positive and negative pairs constructed in this way. For two L2-normalized embeddings **e***_a_* and **e***_b_*, cosine similarity is defined as $s_{\mathrm{ab}}=e_{a}^{⊤}e_{b}$. With margin *m* = 0.25 and scale *γ* = 64, the logits for pairs with the same identity and pairs with different identities are, respectively,

(2) $$l_{\mathrm{ab}}^{\mathrm{pos}}=-\gamma s_{\mathrm{ab}}+\gamma -\mathrm{\gamma m}.$$

(3) $$l_{\mathrm{ab}}^{\mathrm{neg}}=\gamma s_{\mathrm{ab}}-\mathrm{\gamma m}.$$

For anchor *a*, let *P_a_* and *N_a_* denote the positive and negative index sets, respectively. Only anchors with both positive and negative pairs contribute to the loss, giving the valid anchor set 𝒱 = {*a*∣|*P_a_*| > 0∧|*N_a_*| > 0}. For each anchor, the positive and negative logits are aggregated separately and the two terms are added. The softplus function sp(*x*) = log(1 + exp*x*) is applied to this sum, and the results are averaged over valid anchors to obtain the identity loss.

(4) $$\alpha_{a}^{\mathrm{pos}}=\log\sum_{b\in P_{a}}e^{l_{\mathrm{ab}}^{\mathrm{pos}}}.$$

(5) $$\alpha_{a}^{\mathrm{neg}}=\log\sum_{b\in N_{a}}e^{l_{\mathrm{ab}}^{\mathrm{neg}}}.$$

(6) $$\sigma_{a}=\alpha_{a}^{\mathrm{pos}}+\alpha_{a}^{\mathrm{neg}}.$$

(7) $$L_{\mathrm{ID}}=\frac{1}{\left|V\right|}\sum_{a\in V}\mathrm{sp}\;\sigma_{a}.$$

If |𝒱| = 0, the identity loss is set to 0. This cosine-similarity-based identity loss has the same form as the unified pair-similarity objective introduced by Sun et al. [58]. It constructs the loss by separately summing exponential terms for positive and negative similarities, but does not apply the additional similarity-dependent weighting coefficients used in Circle Loss. The term 0.05ℒ_ID_ is added to the overall detector objective. Identity supervision is applied only to the final decoder layer.

ORAT supplies localization quality, as well as identity representations, to the track and detection representations. The localization-quality descriptor is computed deterministically from D-FINE’s final fine-grained distribution refinement (FDR) statistics. Normalized entropy, normalized bin variance, and maximum probability are computed for each of the four bbox edges. The difference between the class logit corrected by localization quality estimation (LQE) and the original class logit is then appended, yielding a 13-dimensional descriptor. This path uses no separate learnable head. Bboxes, detection confidences, identity embeddings, and localization-quality descriptors are aligned by the retrieved query indices and stored together in the cache.

### 3.3. Track State and Observation Relations

Detector-side outputs provide information about observations in the current frame, while track states summarize and retain observations accepted through the previous frame. Rather than relying on a single cumulative summary, ORAT maintains both cumulative track-level state and individual recent observations. Relations with stored observations are recomputed for each current detection candidate, so the same track history can provide different temporal cues for different candidates.

**Cumulative track state.** Each track state includes the last accepted bbox and detection confidence, the number of accepted observations, the current missed-observation gap, a cumulative identity summary, and a cumulative localization-quality descriptor. The confidence supplied to the track encoder is maintained as the mean over accepted observations. The cumulative identity summary is maintained as the arithmetic mean of accepted detector-side identity embeddings. It is initialized when a new track is created, and the mean is subsequently updated only when a match is accepted. This summary is used as input to the track representation without additional L2 normalization; cosine similarity is computed by dividing the inner product of the two vectors by the product of their L2 norms. When a localization-quality descriptor is available for each accepted observation, the cumulative descriptor is maintained as the arithmetic mean over these observations. On unmatched frames, predicted bboxes, zero-valued embeddings, and synthetic appearance information are not written to the cumulative summary or observation memory.

**Recent-observation memory.** The recent-observation memory of track *i* is defined as

(8) $$R_{i}^{t}={\left\{\left(e_{\mathrm{im}},t_{\mathrm{im}}\right)\right\}}_{m=1}^{M_{i}^{t}},\;\;M_{i}^{t}=\left|R_{i}^{t}\right|,\;\;0\leq M_{i}^{t}\leq K_{r}.$$

Here, **e***_im_* is the identity embedding of track *i* corresponding to its *m*th stored observation, and *t_im_* is the frame index at which that observation was stored. $M_{i}^{t}$ is the number of observations in the recent-observation memory (recent memory) at time *t*, with a maximum capacity of *K_r_* = 5. When a new track is created or a match is accepted, only observations with detection confidence of at least 0.75 are stored in FIFO order.

Recent-observation relations are computed from similarity statistics between stored identity embeddings and the current detection, memory occupancy, and observation age. Detection confidence determines whether an observation is stored. Historical bboxes and scores retained as auxiliary information in recent-memory entries are not used to compute the recent-relation vector. The geometric cues in Section 3.4 are computed from the track’s last accepted bbox and the current detection bbox. The localization-quality descriptor is incorporated into the cumulative track representation, not into recent-observation relations.

The identity relation between the current detection candidate *j* and each stored observation *m* in recent memory is computed as the following cosine similarity:

(9) $$r_{\mathrm{ijm}}^{t}=\cos\left(e_{\mathrm{im}},e_{j}^{t}\right).$$

When recent memory is nonempty, the index of the stored observation with the highest similarity is defined as $m_{\mathrm{ij}}^{⋆}={\arg\;\max}_{1\leq m\leq M_{i}^{t}}r_{\mathrm{ijm}}^{t}$. Thus, $m_{\mathrm{ij}}^{⋆}$ refers to the observation whose identity representation is most similar to that of the current detection candidate *j*. Its age cue is computed by dividing the number of frames elapsed since the observation was stored by the maximum missed-observation gap defined in Section 3.5 and capping the result at 1.

(10) $$q_{\mathrm{ij}}^{\mathrm{age}}=\min\;\left(\frac{t-t_{im_{\mathrm{ij}}^{⋆}}}{G_{\max}},1\right).$$

When recent memory is nonempty, the similarities between candidate *j* and stored observations, sorted in descending order, are denoted by $r_{\mathrm{ij}(1)}^{t}\geq r_{\mathrm{ij}(2)}^{t}\geq \ldots \geq r_{\mathrm{ij}(M_{i}^{t})}^{t}$. Parenthesized indices indicate similarity rank. The number of observations used to average the highest similarities is $h_{i}^{t}=\min(3,M_{i}^{t})$. Together with the age cue defined in Equation (10), these quantities form the following five-dimensional recent-observation relation vector:

$$q_{\mathrm{ij}}^{\mathrm{recent}}={\left[\begin{array}{c} r_{\mathrm{ij}(1)}^{t}\\ \frac{1}{h_{i}^{t}}\sum_{u=1}^{h_{i}^{t}}r_{\mathrm{ij}(u)}^{t}\\ r_{\mathrm{ij}(1)}^{t}-r_{\mathrm{ij}(2)}^{t}\\ \frac{M_{i}^{t}}{K_{\mathrm{ref}}}\\ q_{\mathrm{ij}}^{\mathrm{age}} \end{array}\right]}^{T},K_{\mathrm{ref}}=8.$$

If fewer than three observations are stored, the mean of the top 3 similarities is computed using only the $h_{i}^{t}$ observations actually available. If only one observation is stored, the nonexistent second-highest similarity is set to $r_{\mathrm{ij}(2)}^{t}=0$ when computing the difference between the highest and second-highest values. The similarity gap is used without additional clipping. If recent memory is empty, the index of the most similar observation is left undefined, the mean is not computed, and we set $q_{\mathrm{ij}}^{\mathrm{recent}}=[0,\;0,\;0,\;0,\;1]$.

The maximum similarity indicates how closely the most similar stored observation matches the current detection. The mean of the top 3 similarities jointly describes the support provided by the most similar stored observations. The similarity gap is the difference between the highest and second-highest similarities. The occupancy cue conveys the amount of available stored observations, while the age cue conveys the time elapsed since the most similar observation. A larger age cue corresponds to an older observation. Thus, even for the same track memory, recent-relation cues can differ depending on the detection candidate being compared.

The occupancy cue is scaled by the fixed reference *K_ref_* = 8, which is separate from the actual maximum capacity of each memory. This reference maintains compatibility with the input scale used during initial tracker training and is also used for the long-term occupancy cue defined below.

In this paper, ORAT denotes the base configuration using cumulative track state and recent-observation memory, whereas ORAT (LT) denotes an optional extension that adds long-term memory of representative identity embeddings. The capacities of the two memories are specified in Section 4.1.3.

**Optional long-term memory of representative identity embeddings.** ORAT (LT) maintains long-term memory $L_{i}^{t}$ to supplement the recent-observation path. This memory stores up to *K_l_* L2-normalized representative identity embeddings. To create a new entry, the current identity embedding is L2-normalized and stored as a representative embedding with a support count of 1. Its usage and reliability are initialized to the current detection confidence, and its last-used frame is set to the current frame.

If the cosine similarity between a new embedding and the most similar existing representative embedding is at least 0.75, they are combined with weights of 0.9 for the existing representative and 0.1 for the new embedding, followed by L2 normalization. The support count is incremented by 1, and usage is updated as the arithmetic mean of the confidences of observations accepted so far. The representative embedding’s reliability is computed as the mean of its usage and the nonnegative merge similarity. Its last-used frame is updated to the current frame.

If adding a new representative embedding would exceed capacity, entries with higher reliability are retained first. Ties in reliability are resolved by higher usage, followed by a larger last-used frame index if usage is also tied. Only four representative embeddings are retained.

Let $L_{i}^{t}=|L_{i}^{t}|$ denote the number of representative embeddings in long-term memory, and let *n* index a representative embedding $p_{\mathrm{in}}^{t}$ with reliability $\rho_{\mathrm{in}}^{t}$. When long-term memory is nonempty, the similarity between the current detection candidate’s identity embedding and the *n*th representative embedding is computed as $l_{\mathrm{ijn}}^{t}=\cos(p_{\mathrm{in}}^{t},e_{j}^{t})$. The similarities sorted in descending order are denoted by $l_{\mathrm{ij}(1)}^{t}\geq l_{\mathrm{ij}(2)}^{t}\geq \ldots \geq l_{\mathrm{ij}(L_{i}^{t})}^{t}$. Parenthesized indices indicate similarity rank, and the number used for the top-three mean is $v_{i}^{t}=\min(3,L_{i}^{t})$. The index of the most similar representative embedding is $n_{\mathrm{ij}}^{⋆}={\mathrm{arg max}}_{n}l_{\mathrm{ijn}}^{t}$. ORAT (LT) uses the following five-dimensional long-term relation vector:

$$q_{\mathrm{ij}}^{\mathrm{long}}={\left[\begin{array}{c} l_{\mathrm{ij}(1)}^{t}\\ \frac{1}{v_{i}^{t}}\sum_{u=1}^{v_{i}^{t}}l_{\mathrm{ij}(u)}^{t}\\ l_{\mathrm{ij}(1)}^{t}-l_{\mathrm{ij}(2)}^{t}\\ \frac{L_{i}^{t}}{K_{\mathrm{ref}}}\\ \rho_{in_{\mathrm{ij}}^{⋆}}^{t} \end{array}\right]}^{T}.$$

If fewer than three representative embeddings are stored, the mean of the top 3 similarities is computed using only the $v_{i}^{t}$ representative embeddings actually available. If only one representative embedding is stored, the implementation sets the nonexistent second similarity to $l_{\mathrm{ij}(2)}^{t}=0$. The similarity gap is used without additional clipping. If long-term memory is empty, the index of the most similar representative embedding is left undefined, the mean is not computed, and we set $q_{\mathrm{ij}}^{\mathrm{long}}=[0,\;0,\;0,\;0,\;0]$.

The first four components follow the same order as in the recent-relation vector: maximum similarity, top-three mean, similarity gap, and occupancy. They describe the strongest match to the current detection, the support from the highest-similarity representative embeddings, the difference between the two highest similarities, and the number of available representative embeddings, respectively. Occupancy is scaled by the same fixed reference *K_ref_* = 8 used for recent memory; long-term memory retains at most four representative embeddings. The final component is the reliability of the representative embedding most similar to the current detection.

ORAT (LT) uses long-term relations when the track’s missed-observation gap is at least 30 frames. As shown in Equation (11), the gate is applied to the long-term relation vector, which is then concatenated after the recent-relation vector. The long-term bank is maintained according to the storage and update rules for accepted observations described above; the 30-frame condition determines when long-term relations are used in affinity computation.

(11) $$\eta_{i}^{t}=1\left[g_{i}^{t}\geq 30\right],\;\;q_{\mathrm{ij}}^{\mathrm{mem}}=\left[q_{\mathrm{ij}}^{\mathrm{recent}},\eta_{i}^{t}q_{\mathrm{ij}}^{\mathrm{long}}\right].$$

The memory relation is a 10-dimensional vector formed by concatenating five recent-relation components and five long-term-relation components. In ORAT (LT), when $g_{i}^{t}<30$, only the five long-term components are zero, while the five recent components are retained. The base ORAT configuration does not use a long-term bank, regardless of the gap, and always sets the corresponding five input positions to zero. In both configurations, this 10-dimensional memory relation is concatenated with five basic cues to form the 15-dimensional Pair-Bias input in Section 3.4.

Bounding the capacities of recent-observation memory and optional long-term memory keeps the number of observations accessed by each candidate pair within a fixed range as trajectories grow longer. The total cost of computing relations depends on the numbers of active tracks and detections. With identity embedding dimension *d*_id_, relation computation in the base configuration scales approximately as *O*(*N_T_N_D_K_r_ d*_id_); using the long-term path gives a complexity of *O*(*N_T_N_D_*(*K_r_* + *K_l_*)*d*_id_). These expressions describe memory-relation computation; the complete association procedure also includes the costs of the Transformer, Pair-Bias, and assignment.

### 3.4. Structured Affinity and Training Objectives

**Figure 2:**
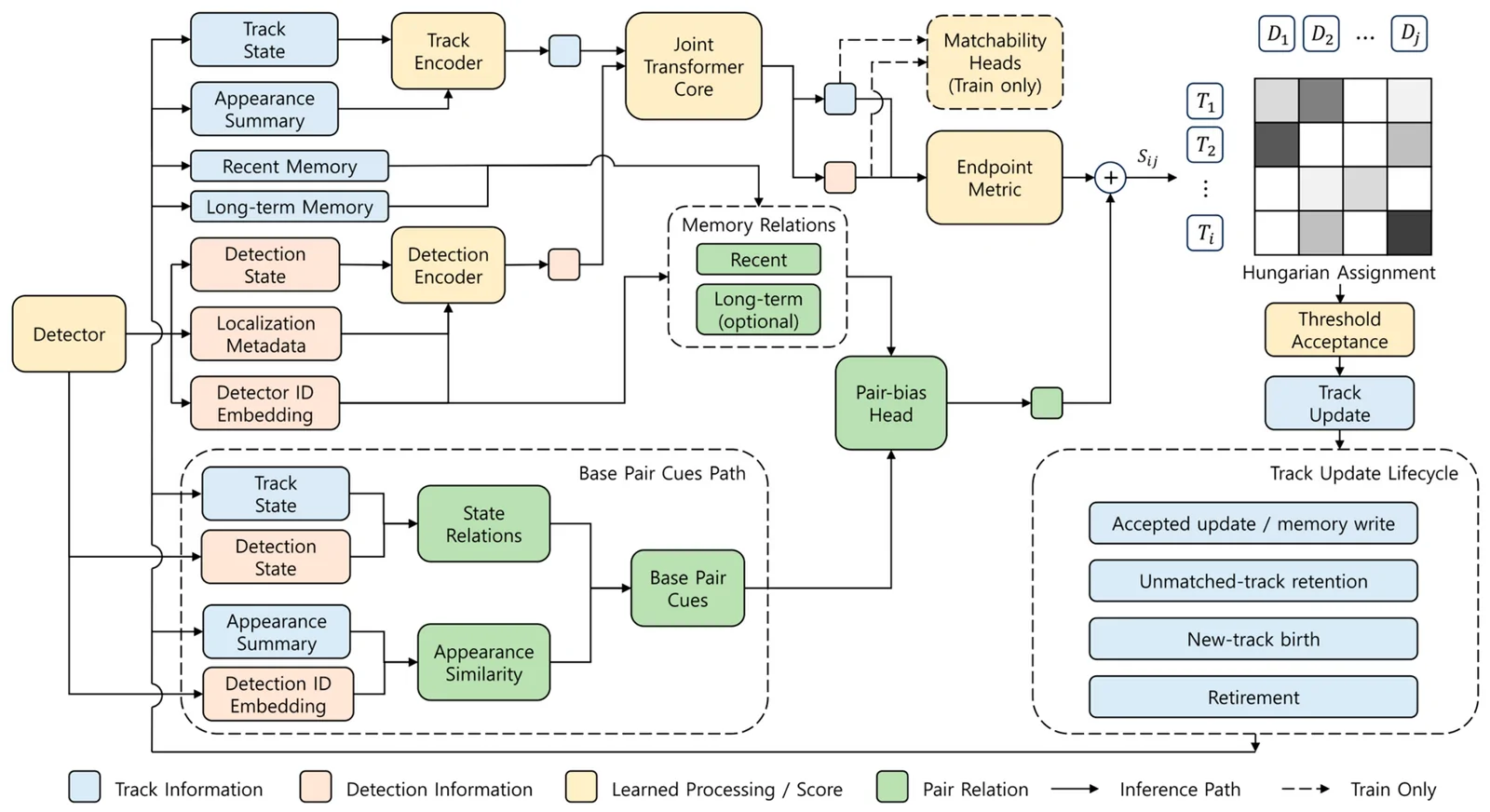
ORAT’s association architecture under online conditions. Detector outputs aligned by query index and cumulative track states are encoded to generate contextualized track and detection representations. The relation path computes relations between the current detection and the track or a bounded set of stored observations. The output of the Pair-Bias Head, which uses these relations, is added to the representation-distance score to obtain an unnormalized affinity score. Pairs satisfying the geometric gate are used to form Hungarian one-to-one assignment proposals. Only proposals that also satisfy the affinity threshold are accepted as final matches for state and memory updates. In the schematic affinity matrix, rows denote tracks (T), columns denote detections (D), and shaded cells represent pairwise affinity scores. Ellipses indicate additional tracks or detections. Abbreviations: ORAT, Observation- and Relation-based Affinity Tracker; ID, identity.

The detector outputs in Section 3.2 are encoded as detection representations, and the track states in Section 3.3 are encoded as track representations. These representations are combined with relations between the current detection and stored observations, geometry, and confidence cues to compute an affinity score for each track–detection pair. Figure 2 shows how detector outputs aligned by query index, cumulative track state, and bounded observation memory are combined into a single association score.

The track encoder receives the last accepted bbox and the mean detection confidence of accepted observations, the number of accepted observations, the missed-observation gap, the cumulative identity summary, and the cumulative localization-quality descriptor. The detection encoder input comprises four normalized coordinates of the current bbox, one detection confidence value, one constant state feature indicating a current detection, a 13-dimensional current localization-quality descriptor, and a 512-dimensional detector-side identity representation. The constant state feature is fixed at 1.0, giving a total detection input dimension of 4 + 1 + 1 + 13 + 512 = 531.

Each track and detection encoder projects its state and identity branches to 128 dimensions each. The identity encoder weights are shared between tracks and detections, while the state encoders and fusion multilayer perceptrons (MLPs) are separate. The branches are concatenated and passed through a fusion MLP to produce a 128-dimensional representation, which is then linearly projected to 32 dimensions. A two-layer Transformer with *d*_model_ = 32 contextualizes these representations jointly with the other active track and detection representations in the same frame. The two layers have independent weights and use four attention heads, a 128-dimensional feed-forward network, GELU, pre-layer normalization, and dropout of 0. The 32-dimensional outputs are L2-normalized without an additional projection to obtain the final track and detection representations.

Let $z_{i}^{\mathrm{trk}}$ and $z_{j}^{\det}$ denote the contextualized track and detection representations, respectively. Their distance-based score is

(12) $$s_{\mathrm{ij}}^{\mathrm{metric}}=-{∥z_{i}^{\mathrm{trk}}-z_{j}^{\det}∥}_{2}^{2}.$$

To add pairwise relation information to the representation-distance score, the Pair-Bias input combines basic cues with observation relations. The five basic cues are IoU, center distance normalized by the image diagonal, the absolute log area ratio, the mean of the track’s average detection confidence and the current detection confidence, and identity cosine similarity. Identity cosine similarity is computed between the track’s cumulative identity summary and the current detection’s identity embedding. The recent-relation vector $q_{\mathrm{ij}}^{\mathrm{recent}}\in R^{5}$ from Section 3.3 and five long-term input components are concatenated with these basic cues to form a 15-dimensional input. ORAT (LT) uses $\eta_{i}^{t}q_{\mathrm{ij}}^{\mathrm{long}}$ from Equation (11). The localization-quality descriptor is used only to generate track and detection representations and is not concatenated directly to this vector. The Pair-Bias Head projects the input to 64 dimensions, applies a rectified linear unit (ReLU), and outputs a single bias score *b_ij_*. The final affinity score is the sum of the representation-distance score and the bias score.

(13) $$S_{\mathrm{ij}}=s_{\mathrm{ij}}^{\mathrm{metric}}+b_{\mathrm{ij}}.$$

The final *S_ij_* is an unbounded, unnormalized affinity score rather than a probability. During training, it serves as the pre-sigmoid logit for the pairwise binary cross-entropy loss; during inference, it is used to construct the cost matrix and apply the affinity threshold.

A cosine-similarity matrix is constructed from the L2-normalized track and detection representations obtained above. Adaptive Circle Loss is applied to its track-wise rows and detection-wise columns [58]. The tracker Circle loss uses margin *m*_trk_ = 0.25 and scale *γ*_trk_ = 32. Stop-gradient is applied to the adaptive weights computed from each similarity, while gradients are allowed to propagate through the similarity used in each logit.

Positive pairs for the Circle loss are defined by ground-truth (GT) identities in the training context and remain positive even when they lie outside the geometric candidate gate. Negative pairs are restricted to pairs inside the gate whose GT identities do not match. A Circle term is computed only for a row or column containing at least one positive and one negative pair; rows and columns that do not meet this condition are excluded from the average.

The track and detection matchability heads are trained with binary cross-entropy (BCE) to predict whether a ground-truth counterpart exists in the training context, regardless of whether it passes the geometric candidate gate. Pairwise BCE uses the final affinity *S_ij_* as its pre-sigmoid logit and averages the losses over positive and negative pairs inside the gate. Both matchability heads are used only during training and do not contribute to affinity scores or assignment during inference.

The final tracker objective is

ℒ_tracker_ = ℒ_circle_ + ℒ_trk-match_ + ℒ_det-match_ + 0.2 ℒ_pair_. (14)

The Circle terms for all rows and columns containing both positive and negative pairs are summed and divided by the total number of valid rows and columns to obtain ℒ_circle_. This loss is set to 0 for a training context with no valid rows or columns. In the complete objective, the Circle term and each of the two matchability BCE terms have a weight of 1, while pairwise BCE has a weight of 0.2.

During tracker training, detector outputs from each sequence were processed in chronological order to construct track states and observation memories. Detections in each frame were matched to GT bounding boxes using IoU-based Hungarian matching, and GT identities were assigned to matches with an IoU of at least 0.5. The states and memories were updated by linking detector observations with the same GT identity rather than using the tracker’s predicted associations. Each training context was constructed before incorporating the current frame’s observations into the states, so the history contained only observations from earlier frames.

Each training context is restricted to a single sequence so that individuals with the same integer ID in different sequences are not mixed into positive pairs. The mini-batch loss is the mean of the losses for the contexts constructed in this way.

### 3.5. Assignment, Acceptance, and Online Lifecycle Management

The affinities computed in the preceding section are used to propose one-to-one correspondences between tracks and detections. ORAT negates the affinity scores of valid candidates to construct the rectangular cost matrix for the Hungarian algorithm. Noncandidate pairs are assigned an affinity score of −10^9^, with the corresponding cost *c*_inv_ = 10^9^ in the cost matrix.

(15) $${\tilde{C}}_{\mathrm{ij}}^{t}=\{\begin{array}{ll} -S_{\mathrm{ij}}^{t}, & C_{\mathrm{ij}}^{t}=1,\\ c_{\mathrm{inv}}, & C_{\mathrm{ij}}^{t}=0. \end{array}$$

If either the track set or the detection set is empty, the assignment set is empty. Otherwise, a rectangular linear sum assignment problem (LSAP) is solved to obtain a set of proposed one-to-one assignments. This problem minimizes the sum of the selected pair costs while assigning each element of the smaller set to a distinct element of the other set.

(16) $$H^{t}=\mathrm{LSAP}\left({\tilde{C}}^{t}\right).$$

The Hungarian algorithm produces one-to-one assignment candidates rather than final matches. ORAT then reapplies the geometric candidate condition and the affinity threshold *τ* selected on validation data to each candidate.

(17) $$A^{t}=\{\left(i,j\right)\in H^{t}|C_{\mathrm{ij}}^{t}=1\wedge S_{\mathrm{ij}}^{t}\geq \tau \}.$$

Pairs whose affinity equals the threshold are also accepted. Assignments below the threshold or failing the geometric candidate condition are treated as unmatched. This is a two-stage procedure: learned affinities first produce one-to-one assignment candidates, and only those satisfying an absolute affinity criterion are accepted as final matches. It therefore differs from directly solving an optimal partial-matching problem with the threshold constraint included in the objective.

Accepted matches update the track’s bbox, detection confidence, number of accepted observations, cumulative identity summary, and cumulative localization-quality descriptor. Recent-observation memory is handled according to the storage and update rules in Section 3.3. Storage and updates of long-term representative embeddings apply only to the ORAT (LT) configuration.

Unmatched tracks retain their last accepted state while their missed-observation gap increases. If a match between the track and a detection is accepted again within *G*_max_ = 180 frames, the existing track ID is retained. The track is terminated once this gap is exceeded.

A new track is created for any detection that passes the tracker-input confidence criterion but is not accepted as a match to an existing track. Tracks lasting only one frame are included in the evaluation output; short tracks are not suppressed separately.

During inference, ground-truth pairs outside the center-distance gate are excluded from the final matching candidates. Incorrect pairs may still be accepted within the gate, and such matches can contaminate the cumulative state, recent-observation memory, and optional long-term memory, which are updated from accepted matches. In all experiments, the candidate gate, affinity threshold, and lifecycle settings were selected on validation data and fixed before evaluation outputs were generated. The experimental conditions, including input filtering, are described in Section 4.1.3.

## 4. Experimental Evaluation

We evaluated ORAT on the PigTrack test split [11] and through two-direction outer-test evaluation on Jochiwon23, separated from internal model selection, using shared detections for the local tracker comparisons. Component experiments examined the effects of detector-side identity representations and recent-observation relations relative to a common baseline. We measured model complexity and closed-loop runtime separately to assess accuracy–throughput trade-offs on server and edge devices. Separate training and evaluation on SportsMOT and DanceTrack assessed the architecture’s applicability to human tracking under different motion and occlusion conditions [22,23]. Qualitative examples illustrate identity continuity in pig videos.

### 4.1. Experimental Protocols, Data, and Reporting Conventions

#### 4.1.1. Dataset Roles and Splits

PigTrack was the primary public benchmark for pig tracking [11]. Jochiwon23 provided supplementary outer-test evaluation using temporally separated video blocks from the same closed herd. In each direction, three sequences were used for training, one for internal checkpoint and affinity-threshold selection, and the other four for outer testing, as detailed in Section 4.1.2. SportsMOT and DanceTrack were used to assess ORAT under motion and occlusion conditions different from those in pig videos, with separate training on each dataset [22,23]. Public dataset statistics and official splits followed the original sources. Table 1 summarizes the scale and evaluation role of each dataset.

The PigTrack detector and tracker were trained only on the official training split. To generate tracker training samples, we divided the official training split into two folds at the sequence level, keeping all frames of each sequence in the same fold. Detections and aligned identity embeddings for each fold were generated by a D-FINE-X detector trained only on the opposite fold. These two-fold out-of-fold (OOF) outputs were used to construct tracker training samples, so each sequence was processed by a detector that had not been trained on that sequence. The tracker checkpoint and affinity threshold were selected using HOTA on the official validation split. After fixing these settings, we evaluated the models on the official test split. Test results were not used to select the number of epochs, threshold, seed, or architecture.

For validation and testing in the main PigTrack experiment, the tracker trained on OOF observations was paired with a separate D-FINE-X detector trained on the entire official training split. The fold-specific detectors and the final detector used the same architecture and feature output format, and no additional embedding alignment or tracker retraining was performed for this pairing. Fold-specific detector checkpoints were selected using detection average precision (AP) on the corresponding held-out folds, whereas the final detector checkpoint was selected using detection AP on the official validation split.

#### 4.1.2. Jochiwon23 Acquisition, Annotation, and Two-Direction Outer Evaluation

Jochiwon23 is a noninterventional, closed-herd dataset selected retrospectively from videos recorded by a fixed overhead camera at a commercial pig farm. This study involved no new animal handling, treatment, or experimental intervention. The eight sequences were recorded from 17:00 on 13 August 2013 to 18:00 on 14 August 2013 and contain 23 pigs. No pigs entered or left the herd during this period. Consecutive sequences are separated by at least five minutes. Identity numbers apply only within each sequence; the same number in different sequences does not necessarily refer to the same pig.

**Figure 3:**
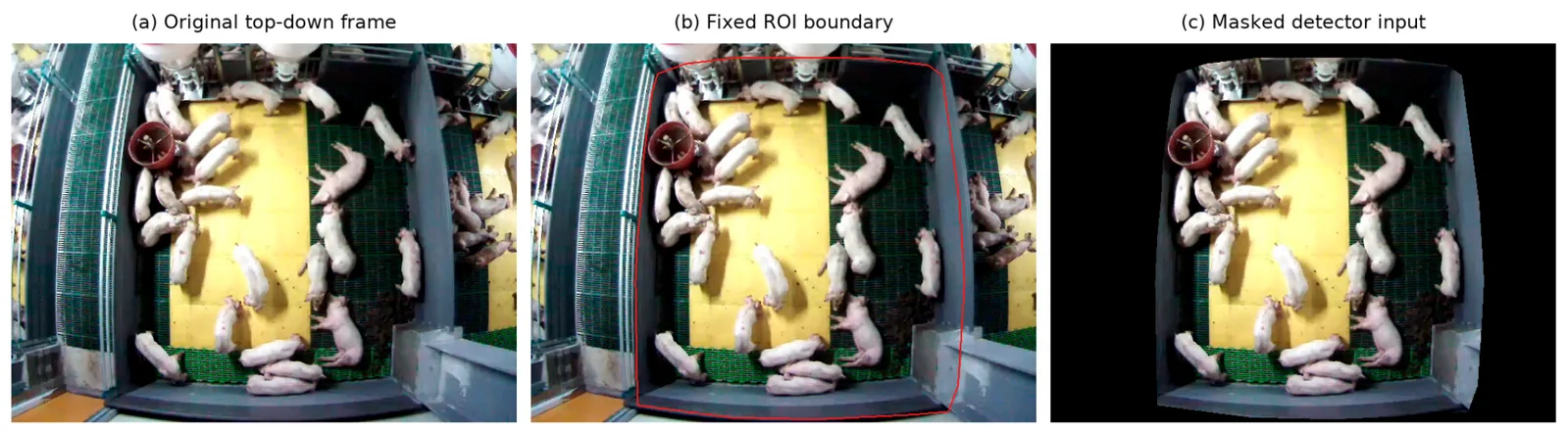
Representative overhead scene from Jochiwon23 and fixed region of interest (ROI) processing: (**a**) original frame, (**b**) ROI boundary shared by all sequences, and (**c**) detector input with pixels outside the ROI set to zero. The red outline marks the ROI boundary.

The evaluation contains 23,867 frames and 546,734 bounding boxes, with a mean of 22.908 pigs per frame. The videos have a resolution of 640 × 480 and were encoded at 25 FPS. We applied one fixed region of interest (ROI) to all sequences and set pixels outside the ROI to zero. The same mask was used during detector training and inference. The masked videos were stored in advance to avoid repeated preprocessing. Figure 3 shows the original scene, the ROI boundary, and the detector input.

Two annotators each labeled one block, Video01–Video04 or Video05–Video08, following the same example-based guidelines. The 23 pigs visible in the first frame of each sequence were assigned arbitrary identity numbers from 1 to 23, which were maintained within that sequence. Each annotator then checked the other block. Ambiguous identities were reviewed using preceding and subsequent frames, corrected when necessary, and checked again. The first author inspected all sequences in temporal order and corrected remaining identity inconsistencies using the same procedure. Pigs outside the pen, reflections, and facility structures were excluded. Inter-annotator agreement was not calculated separately.

Jochiwon23 was evaluated in two directions, separating internal model selection from outer evaluation. In direction A, models were trained on Video01–03, the checkpoint and affinity threshold were selected on Video04, and outer evaluation was performed on Video05–08. In direction B, models were trained on Video05–07, settings were selected on Video08, and outer evaluation was performed on Video01–04. The outer-test block in each direction was not used to train or select the detector, external ReID model, or tracker. After the selected settings were fixed, outer-test outputs from both directions were evaluated jointly with TrackEval 1.0.dev1 while preserving sequence boundaries. The result was computed across all 8 outer-test sequences rather than as the arithmetic mean of the directional HOTA scores. Although evaluation used videos excluded from model selection, the videos were still acquired from the same farm and herd, so this protocol did not evaluate generalization across farms or individual animals. Related studies provide context for the recording environment but do not use the same 8 sequences as this study [43,59].

#### 4.1.3. Training and Inference Settings

The main D-FINE-X input resolutions are 1280 × 800 for PigTrack and 640 × 480 for Jochiwon23. The final detector query is 256-dimensional, and the Query ID Head generates a 512-dimensional identity embedding. The association model uses two Transformer layers and four attention heads. The main ORAT results reported in Section 4.2 and Section 4.5 use the base configuration combining detector-side identity, recent memory, auxiliary matchability, and localization quality. Recent-observation memory stores up to *K_r_* = 5 observations, and the base configuration does not use a long-term memory bank. The optional ORAT (LT) extension additionally retains up to *K_l_* = 4 representative identity embeddings and uses long-term relations when the missed-observation gap is at least 30 frames. Both configurations use a maximum missed-observation gap of *G*_max_ = 180 frames. Candidate pairs are retained when their center distance, normalized by the image diagonal, is at most 0.35. The IoU lower bound was set to 0.0, with no additional IoU-based exclusion rule. In the server experiments on PigTrack and Jochiwon23, the confidence floor was 0.05 for detection cache generation and 0.75 for ORAT input. SportsMOT and DanceTrack use an input resolution of 1280 × 736.

Trackers were trained using AdamW, a learning rate of 5 × 10^−4^, weight decay of 10^−4^, and a context batch size of 8. Training lasted 20 epochs on PigTrack and in each evaluation direction on Jochiwon23. Jochiwon23 checkpoints and affinity thresholds were selected on the internal validation sequence for each direction. SportsMOT and DanceTrack models were trained independently. For each dataset, validation HOTA from a model trained on the training split was used to select the training epoch and affinity threshold. With these settings fixed, the model was retrained on the combined training and validation splits to generate test results. Test results were not used for model selection.

Temporal intervals and history lengths were not separately sampled at random. The temporal span covered by recent memory may vary with the actual frame intervals between stored observations. Training mini-batches were formed by sampling frame-level contexts constructed in chronological order.

The D-FINE-X model was trained for 36 epochs with AdamW. The base learning rate was 2.5 × 10^−4^, the backbone learning rate was 2.5 × 10^−6^, weight decay was 1.25 × 10^−4^, and the total batch size was 16. We used automatic mixed precision and a maximum gradient norm of 0.1, without warm-up.

#### 4.1.4. Comparison Categories, Evaluation Metrics, and Statistical Reporting

We report HOTA, detection accuracy (DetA), Association Accuracy (AssA), Identification F1 Score (IDF1), and Multiple Object Tracking Accuracy (MOTA), in that order. DetA measures detection performance based on matched, missed, and false detections. AssA measures how well observations of the same identity are linked and observations of different identities are kept separate. HOTA averages the geometric mean of DetA and AssA over multiple bounding-box overlap thresholds and is the primary metric in this study [60]. IDF1 is the harmonic mean of identity precision and recall after global one-to-one matching between ground-truth and predicted identities [61]. MOTA summarizes missed detections, false detections, and identity switches relative to the number of ground-truth detections [62].

Training Scope describes how the target dataset was used to optimize learnable parameters in the complete system, including pretrained detectors and ReID models. T indicates training on the target training split, with validation data used only for checkpoint, threshold, or calibration selection. T + V indicates that validation samples were also used for parameter optimization. N/A denotes no training on the target dataset, and N/R indicates insufficient information to determine the training scope. A tracker is not marked N/A solely because its association module requires no training if its detector or ReID model was pretrained on the target dataset.

In the Appearance column, — indicates no learned appearance representation, E indicates an external appearance path based on bbox crops, and I indicates identity representations generated within a detector, query-based model, or end-to-end model. A ✓ in Extra indicates the use of external data, pose, depth, keypoints, or additional task-specific supervision. The PigTrack results distinguish local comparisons using the same detections from published full-system results. In the PigTrack and Jochiwon23 comparisons, † denotes local reproduction results obtained using a common D-FINE-X detector and detection protocol. In the server comparisons on PigTrack and Jochiwon23, the external baselines received outputs from the common D-FINE-X detector with a confidence floor of 0.05 and top-k set to 300. Subsequent handling of high- and low-confidence detections and the rules for initiating new tracks followed each tracker’s official implementation. For the FTO-SORT comparison, the hyperparameter configuration was selected on validation data and then held fixed. ORAT took as input only those bounding boxes from the same detector that had confidence scores of at least 0.75. Following the original method, FTO-SORT was used without external ReID and connected to the common D-FINE-X detector in place of its original YOLO detector [43]. FTO-SORT’s test accuracy and E2E speed were measured using outputs generated by live detector inference. Final DetA can differ because of tracker-specific filtering and lifecycle rules. Published results include the detectors, training scopes, appearance representations, and lifecycle settings of their respective sources and are therefore presented separately from local comparisons.

Single test results are reported as point estimates for the selected models. HOTA in the common-baseline component experiments is reported as the mean ± sample standard deviation (SD) across five fixed training seeds, 0–4. The identity-representation and recent-observation readout controls use training seed 0, while the gate-sensitivity evaluation varies only the gate for the same selected model. These accuracy evaluations are distinct from the multiple-training-seed analyses in Section 4.3.2 and Section 4.5.2. Runtime is reported as the mean ± sample SD across five runs of a representative model, distinct from variation across training seeds. Higher accuracy and FPS and lower latency indicate better performance. The best value in each comparison is bold, and the second-best is underlined; rankings use unrounded values when available. Downward arrows for parameters and neural computation in billions of floating-point operations (GFLOPs) indicate that smaller model size and lower neural computation are preferred, respectively.

Uncertainty in the main HOTA differences was analyzed using a sequence-level paired bootstrap. In each of 10,000 resampling iterations, the same sequence sample was applied to both comparison conditions, HOTA was reaggregated for that sample, and the 2.5th and 97.5th percentiles of the differences were used as the 95% confidence interval (CI). The bootstrap random seed was 59,001. For multi-seed comparisons, the sequence multiplicities in each bootstrap sample were applied identically to all five seeds, and the mean of the seed-specific HOTA differences was calculated. Frames were not resampled as independent observations, and models were not retrained in each bootstrap iteration. The SportsMOT validation analysis is conditional on the selected models. A supplementary source-cluster analysis using 13 original video groups as resampling units is reported separately from the sequence-level analysis.

#### 4.1.5. Measurement Boundaries for E2E and Tracker-Only Runtime

End-to-end (E2E) runtime covers the full pipeline from input-frame decoding to the final synchronized track update. The measurement scope includes decoding, resizing, host-to-device transfer, detector inference, identity and localization descriptor generation, post-processing, feature transfer, association, linear assignment, lifecycle management, and synchronization. Configurations using external ReID also include bbox cropping and the corresponding model forward pass. Model initialization, warm-up, result storage, and metric computation are excluded, and E2E FPS is measured directly over this full scope.

Tracker-only latency measures the closed-loop association cost after cached detector outputs are available. It includes candidate construction, neural affinity estimation, masking, linear assignment, acceptance, memory updates, and lifecycle management. The numbers of active tracks, retained detections, candidate pairs, and assignment entries, as well as memory state, can differ across configurations. Latency therefore reflects the workload actually incurred by a configuration rather than the independent additive cost of a single module.

Server measurements used an NVIDIA RTX PRO 6000 Blackwell Workstation Edition graphics processing unit (GPU; NVIDIA Corporation, Santa Clara, CA, USA), an AMD Ryzen 9 9950X central processing unit (CPU; Advanced Micro Devices, Inc., Santa Clara, CA, USA), 60 GiB random-access memory (RAM), Ubuntu 24.04.4 long-term support (LTS), Python 3.12.3, PyTorch 2.11.0+cu130, CUDA 13.0, and cuDNN 9.19. Each measurement was repeated five times in 32-bit floating-point precision (FP32) with batch size 1 after a 50-frame warm-up. Accuracy was computed from the designated evaluation outputs. Jetson comparisons using D-FINE-N at 640 × 640 and TensorRT 10.3 with 16-bit floating-point precision (FP16) are presented in Section 4.4.2. Because the server and Jetson use different detectors and runtimes, accuracy and throughput are compared within each device.

### 4.2. Main Results on Pig Videos

Local comparisons on PigTrack and Jochiwon23 used the same detector outputs to evaluate each tracker’s tracking accuracy and E2E throughput. PigTrack provides a test evaluation on a public benchmark, whereas Jochiwon23 provides two-direction outer evaluation on commercial pig-farm videos, separated from internal model selection.

#### 4.2.1. PigTrack

Table 2 compares tracker association, filtering, and lifecycle rules using identical D-FINE-X detections. Published results use the detectors and training settings of their original sources and are presented separately. Values for the 8 published baselines other than SIPTrack were taken from the PigTrack benchmark [11]. The main ORAT result uses the base configuration combining detector-side identity (ID), recent memory (RM), auxiliary matchability (AM), and localization quality (LQ), as detailed in Section 4.3.2. The affinity threshold *τ* = −10.4, selected on validation, was fixed for test evaluation. Results for the optional long-term extension are reported separately as ORAT (LT).

ORAT achieved the highest HOTA, AssA, and IDF1 in the local shared-detection comparison, at 88.1, 89.5, and 94.9, respectively. Compared with DiffMOT, DetA was similar while AssA and IDF1 were higher, indicating that the differences with the same detector outputs arose primarily in identity association. The identity-representation and recent-observation readout controls below separately analyze design factors related to these association differences.

ORAT achieved an E2E throughput of 48.615 ± 0.035 FPS, approximately 1.56 times that of DiffMOT with external ReID. ByteTrack and OC-SORT were faster but had lower HOTA and association metrics. Generating identity features from detector queries avoids the additional appearance-extraction cost of bbox cropping and a separate ReID forward pass.

Without external ReID, FTO-SORT achieved HOTA of 83.8 and throughput of 45.576 ± 0.138 FPS. ORAT achieved 4.2 points higher HOTA and higher throughput than FTO-SORT, showing improvements in both accuracy and throughput compared with a tracker developed for pig datasets.

#### 4.2.2. Jochiwon23

Table 3 summarizes the Jochiwon23 outer-evaluation results. In each outer-evaluation direction on Jochiwon23, all local methods received the same detections from 640 × 480 videos. Checkpoints and affinity thresholds were selected from the validation results of each condition and fixed before outer-test evaluation. Outputs from both directions were evaluated jointly while preserving sequence boundaries.

ORAT achieved HOTA of 79.1, AssA of 72.6, and IDF1 of 84.9 in outer evaluation. These values were 0.871, 1.831, and 1.612 points higher than those of DiffMOT, respectively, showing differences in association metrics with the same detections in dense videos containing an average of 22.9 pigs per frame. ORAT (LT) achieved the same accuracy metrics and identity switches (IDSW) as the base configuration. FTO-SORT had lower HOTA at 70.9, but fewer IDSW events: 217 compared with 274 for ORAT.

ORAT achieved an E2E throughput of 66.103 ± 0.177 FPS, exceeding the video frame rate of 25 FPS and reaching approximately 1.58 times DiffMOT’s 41.778 ± 0.118 FPS. FTO-SORT was faster than ORAT at 71.014 ± 0.090 FPS but had lower HOTA, AssA, and IDF1. ORAT (LT) achieved 64.720 ± 0.254 FPS without further improvements in the accuracy metrics.

### 4.3. Component and Mechanism Analysis on Pig Videos

Appearance representations and temporal components were analyzed in separate experiments. Section 4.3.1 compares five identity-representation conditions within the base association architecture, while Section 4.3.2 evaluates individual components and their combination against a common baseline. The additional readout control changes the relation inputs while retaining the memory-storage and cumulative-state update rules. These experiments compare different factors and should not be interpreted as a single sequential ablation.

#### 4.3.1. Appearance Representation Interfaces

Five identity-representation conditions, A, B, C, D, and E, were compared. A uses the 256-dimensional final queries of a detector trained without identity loss. B and C share the same detector and bbox outputs and use 256-dimensional pre-head queries and 256-dimensional Query ID Head outputs, respectively. D and E use the same detections with 512-dimensional OSNet-x1.0 features extracted from bbox crops and 512-dimensional identity embeddings from the detector’s ID head, respectively [21]. All conditions use the base architecture, with a recent-memory capacity of 5, a running-mean identity state, and an inactive long-term bank. A tracker was trained for each representation using seed 0, and its checkpoint and affinity threshold were selected on validation.

The OSNet-x1.0 model used for the final PigTrack evaluation was initialized with ImageNet-pretrained weights and then trained for pig identity recognition using crops extracted from detector bounding boxes in the official training split. The checkpoint was selected using retrieval mean average precision (mAP) on the official validation split. Input crops were converted to red–green–blue (RGB), resized to 256 × 128 (height × width), and normalized using the ImageNet mean and standard deviation.

Tracking accuracy in Table 4 was computed in a controlled evaluation using stored detections and features, while E2E FPS was obtained from separate live-pipeline measurements using the same selected configurations. For standalone retrieval analysis, actual detections with confidence of at least 0.75 were matched to ground truth using Hungarian assignment, and pairs with IoU below 0.5 were excluded. For each identity within a sequence, the first observation served as the query and the last observation at least 100 frames later served as the gallery sample. The entire test-split gallery was searched using cosine similarity between L2-normalized features.

The sequence-level paired bootstrap described in Section 4.1.4 was applied to the main test HOTA differences in Table 4. In the matched 256-dimensional comparison, Query ID Head output C achieved 0.901 points higher test HOTA than pre-head query B, with a 95% CI of [0.597, 1.301]. The CI was entirely above zero with the detector and dimensionality held constant, supporting the benefit of using representations after the dedicated head. The difference between C and query A from a detector trained without identity loss was +0.352 points, with a 95% CI of [−0.245, 1.064], which included zero.

In the 512-dimensional comparison, detector-side embedding E achieved 0.775 points higher cache-based test HOTA than external OSNet D, with a 95% CI of [0.171, 1.357]. Their E2E throughputs were 48.615 ± 0.035 FPS and 33.967 ± 0.047 FPS, respectively, giving E approximately 1.43 times the throughput of D, an increase of 43.1%. External OSNet achieved a higher Rank-1 score, at 43.516% compared with 22.857% for E, whereas the ranking by tracking HOTA was reversed. Rank-1 evaluates whether the top-ranked retrieval has the correct identity, while ORAT uses identity features together with geometry, localization information, and recent-observation relations. Standalone retrieval performance therefore does not by itself determine tracking accuracy. The detector-side path provides identity features without bbox cropping or a separate ReID forward pass.

#### 4.3.2. Common-Baseline Component Experiments on PigTrack

Table 5 compares the state–geometry baseline, the ID baseline with detector-side identity, and configurations that separately add recent memory, auxiliary matchability, or localization quality to the ID baseline. These three additions are not sequentially accumulated. The base ORAT configuration combines ID, RM, AM, and LQ, whereas ORAT (LT) additionally includes the long-term path. HOTA is reported as the mean ± sample SD across training seeds 0–4. Validation and test results were evaluated over the full frame range of each sequence. Tracker latency is reported as the mean ± sample SD of five closed-loop runs of each seed 0 model on the PigTrack test.

Adding the recent-memory configuration to the ID baseline increased mean validation HOTA from 94.148 to 94.599, a gain of 0.452 points. This was the largest increase among the three independent additions to the ID baseline. However, enabling memory in this comparison changes not only individual-observation storage and relation readout but also the identity state, from the last observation to a cumulative mean. The entire change in Table 5 is therefore not interpreted as the effect of readout alone; a separate control retaining the storage and update rules is presented in Table 6. The mean changes from auxiliary matchability and localization quality were smaller than that from recent memory. ORAT, combining all four components, achieved the highest mean validation HOTA at 94.622 ± 0.024.

#### 4.3.3. Auxiliary Long-Term Path

ORAT (LT) adds a long-term bank containing a bounded number of representative identity embeddings to the base configuration. The main comparisons consistently use the base configuration, with LT presented as an optional extension. The base configuration was selected because it achieved comparable multi-seed validation performance to ORAT (LT) on PigTrack and SportsMOT, while requiring less computation.

On PigTrack, mean validation HOTA was 94.622 for the base configuration and 94.606 for LT. The test means showed the opposite ordering: LT achieved 88.106, exceeding the base configuration’s 88.052 by 0.053 points. However, the paired bootstrap 95% CI for the base-minus-LT difference was [−0.153, 0.048], which included zero. LT also had higher tracker latency at 3.221 ± 0.008 ms/frame. We therefore report LT separately rather than concluding that it is always beneficial or detrimental. Given the small changes on PigTrack, the same multi-seed configuration comparison was also performed on SportsMOT.

#### 4.3.4. Recent-Observation Relation Readout

To distinguish the role of recent-observation relations from changes in storage and state updates, we varied the readout supplied to Pair-Bias while retaining the memory bank and running-mean identity state. The long-term bank was disabled in all conditions. R0 uses no relation readout. R1 uses the maximum similarity, the mean of the top $\min(3,M_{i}^{t})$ similarities, and the gap between the highest and second-highest similarities. R2 uses occupancy and the age of the observation most similar to the current detection, whereas R3 uses all five cues. Because R2 also selects the observation for its age cue using the similarity argmax, it is not an appearance-free control. Each condition used training seed 0 and validation-based selection; only E2E runtime was measured over five repeated runs. R0 also omits unnecessary readout computation, so computational work was not held exactly constant across the four conditions.

R3 achieved 1.141 points higher test HOTA than R0 without readout, with a paired bootstrap 95% CI of [0.377, 1.863]. The difference from R2 was +0.724 points, with a CI of [0.193, 1.260]. These results support the benefit of supplying per-candidate relation readouts to association rather than only retaining stored state.

However, R1, using only similarity cues, achieved test HOTA of 88.370, higher than 88.064 for R3 with all cues. The R3 − R1 difference was −0.306 points, with a 95% CI of [−0.647, −0.048]. Although R3 was selected by validation HOTA, R1 achieved higher test HOTA. Thus, the results do not indicate that adding occupancy and age is always beneficial or that each of the five cues is essential.

#### 4.3.5. Candidate-Gate Sensitivity

To examine the effect of the geometric candidate gate, we fixed the checkpoint and affinity threshold of the base seed 0 model on PigTrack validation and varied only the upper bound on center distance normalized by the image diagonal over 0.25–0.45. The checkpoint was from epoch 6, and the affinity threshold was −10.4. Table 7 summarizes the resulting gate-sensitivity measurements.

Positive-pair recall was defined as the proportion passing the gate among pairs for which both a current detection matched to the ground truth and an alive, eligible track of that ground-truth identity existed. The 1723 cases without a ground-truth detection match and the 3726 cases without a corresponding alive track were excluded from the denominator of this conditional recall. This metric therefore differs from detector recall or recall over all ground-truth trajectories.

Conditional positive-pair recall was 100% at all evaluated gates, and HOTA was nearly identical at 94.608–94.609. Widening the gate from 0.25 to 0.45 increased the mean candidate count from 183.6 to 334.0, while tracker latency remained similar at approximately 2.66 ms/frame. In the current implementation, the Transformer processes all track and detection tokens, and Pair-Bias computes the full track–detection matrix. Reducing the number of assignment candidates therefore does not reduce neural computation proportionally. The gate of 0.35 used in this study lies within this range of stable accuracy and conditional recall. The nearly identical results at neighboring gate values support using 0.35 as a reference setting that is not sensitive to a particular boundary value.

### 4.4. Computational Cost and Accuracy–Throughput Trade-Offs in Deployment

#### 4.4.1. Complexity and Server Runtime

Table 8 distinguishes parameter counts, neural GFLOPs, and measured latency for base ORAT. Parameter counts include unique parameters registered in the deployed objects; the 4354 parameters of the training-only auxiliary matchability head are removed from the deployed model and excluded. The count also includes 513 inactive parameters registered in the detector. The detector, Query ID Head, and association tracker therefore contain 61,540,975, 197,888, and 257,487 parameters, respectively, for a total of 61,996,350.

Neural floating-point operations (FLOPs) are calculated using 1 MAC = 2 FLOPs for convolutions and matrix multiplications, with normalization, activation, softmax, bias, and non-matrix attention operations added according to explicit elementwise approximation conventions. Neural GFLOPs approximate computation within this neural-operation scope. The costs of memory-relation computation using NumPy 2.4.4, candidate-gate construction, LSAP, and lifecycle operations are evaluated as part of tracker latency. MAC denotes a multiply–accumulate operation.

Each latency is measured independently at its specified input and output boundaries. The detector path includes GPU transfer of prepared inputs, D-FINE-X inference, localization descriptor output, and detection post-processing, but excludes the Query ID Head. The ID-head measurement covers conversion of [1,300,256] final queries into [1,300,512] L2-normalized embeddings. Independently measured module latencies were 16.324 ms/frame for the detector and 0.050 ms/frame for the ID head. Tracker and full-pipeline latencies are reported as the mean ± sample SD across five runs of the base configuration within their respective measurement scopes.

Association-tracker latency includes candidate construction from detections and memory, neural affinity estimation, masking, linear assignment, threshold acceptance, memory and track-state updates, and lifecycle management. The Added modules row reports the summed parameters and GFLOPs of the ID head and association tracker. Latencies are reported in the rows corresponding to their respective measurement boundaries. Full pipeline is measured directly from input-frame decoding and resizing to the final synchronized track state.

The Query ID Head and association tracker add 455,375 parameters and 0.135 GFLOPs/frame, accounting for approximately 0.735% of total parameters and 0.027% of neural computation. Reusing detector queries keeps the neural computation required for learned identity and association small. Most neural computation occurs in the detector, making detector size an important determinant of overall throughput.

Across the 8939 frames of PigTrack test, association-network computation averaged 0.016 GFLOPs/frame, with p95 of 0.022 GFLOPs/frame. The mean numbers of active tracks and detections per frame were 21.16 and 19.34, respectively. The mean number of valid candidate pairs was 297.40, with p95 of 555. Computation was calculated from the actual track and detection shapes for each frame and then averaged with frame-count weighting. The Transformer processes all active-track and detection tokens, and Pair-Bias computes the full track–detection matrix. The network computes affinity for all track–detection pairs, and valid candidates passing the gate are used for assignment. Tracker latency, including candidate construction and linear assignment, was 2.788 ± 0.008 ms/frame, and full-pipeline latency was 20.570 ± 0.015 ms/frame.

#### 4.4.2. Jetson Orin Nano Super and Accuracy–Throughput Trade-Offs

The edge comparison in Table 9 uses a separately trained D-FINE-N detector at 640 × 640 and the same ORAT tracker interface. Jetson Orin Nano Super measurements were performed in MAXN_SUPER mode with Ubuntu 22.04.5, L4T R36.4.3, CUDA 12.6, TensorRT 10.3, cuDNN 9.3, and PyTorch 2.8.0+cu126. Each method processed the same 8939 PigTrack test frames in the same order, with detector and external ReID engine batch sizes of 1 and 8, respectively. The full interval was measured five times, each after a 30-frame warm-up; cooling time between runs was excluded. The measurement path also included common MOT-result formatting before final synchronization.

On Jetson, the base ORAT configuration achieved HOTA of 74.1 and E2E throughput of 16.3 FPS, with a throughput sample SD of 0.1 FPS across 5 measurements. Its HOTA was slightly lower than DiffMOT’s, but its throughput was approximately 2.75 times that of DiffMOT; both HOTA and throughput were higher than those of the other external ReID methods in the comparison. FTO-SORT was faster than ORAT at 18.4 FPS, but ORAT achieved 4.1 points higher HOTA. The base configuration also achieved higher HOTA and throughput than the optional ORAT (LT) extension, supporting its use in this edge environment. These results show that the design can support both tracking accuracy and throughput without separate ReID inference.

**Figure 4:**
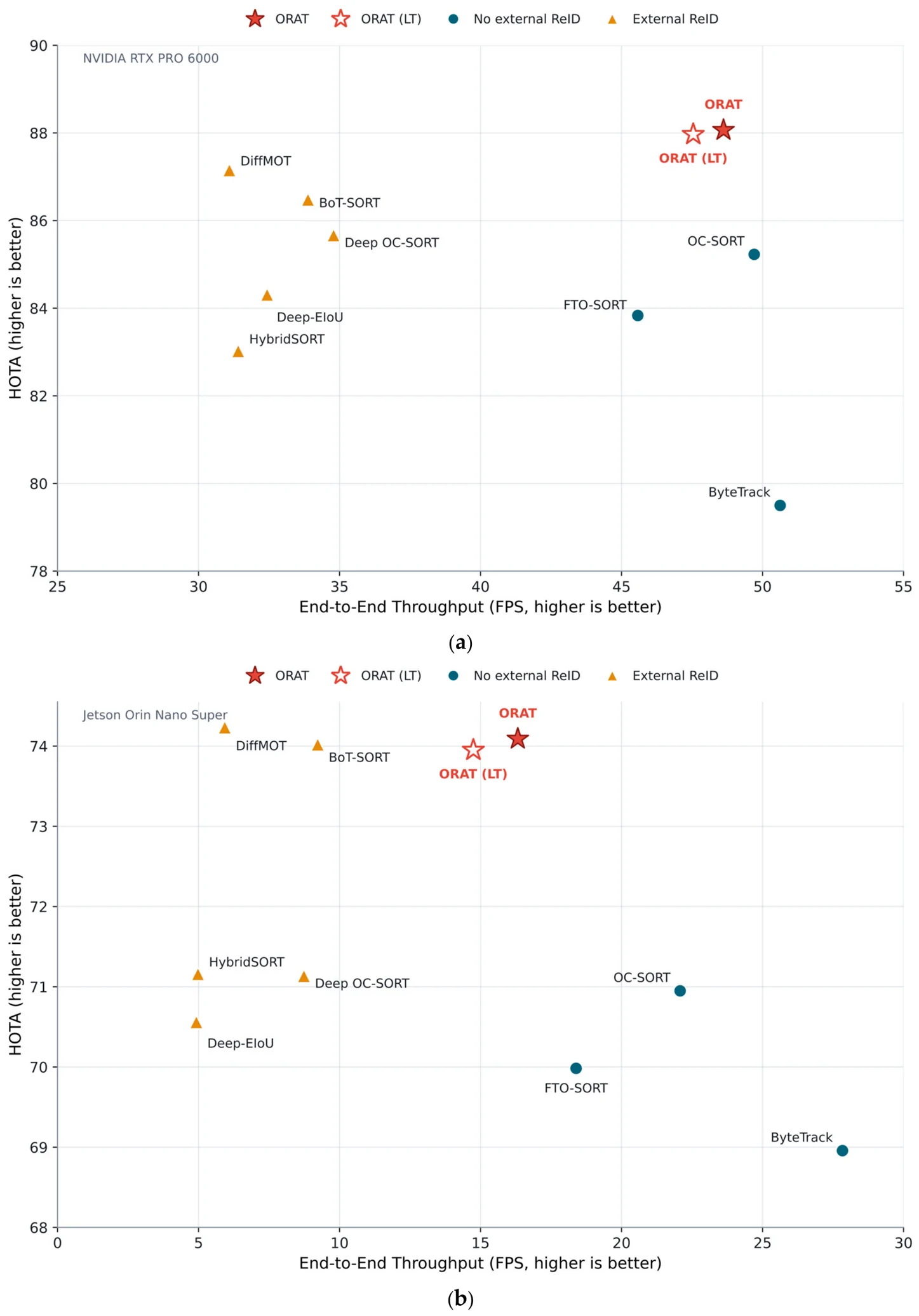
Full-pipeline accuracy–throughput comparison on PigTrack. (**a**) Server results using D-FINE-X and PyTorch FP32. (**b**) Jetson Orin Nano Super results using D-FINE-N and TensorRT FP16. ORAT denotes the base configuration, and ORAT (LT) denotes the optional long-term extension. Because the detectors and runtimes differ, measurement points are compared within each panel. Abbreviations: ORAT, Observation- and Relation-based Affinity Tracker; LT, long-term; HOTA, Higher Order Tracking Accuracy; FPS, frames per second; FP32/FP16, 32-bit/16-bit floating-point precision.

Figure 4a compares full-pipeline accuracy and throughput on the PigTrack server evaluation. Base ORAT achieved HOTA of 88.1 and 48.6 FPS, exceeding the local external ReID methods in both HOTA and throughput. ByteTrack and OC-SORT were faster but had lower HOTA. FTO-SORT, which does not use external ReID, achieved HOTA of 83.8 and 45.6 FPS. The measurement point for the optional ORAT (LT) extension is also shown separately. In the Jetson comparison in Figure 4b, ORAT again achieved higher throughput than external ReID methods and higher HOTA than ByteTrack, OC-SORT, and FTO-SORT. Because the devices use different detectors and runtimes, methods are compared within each panel.

### 4.5. Supplementary Evaluation on Public Human-Tracking Videos

SportsMOT and DanceTrack extend the evaluation beyond pig videos. ORAT was trained separately on each target dataset, so these experiments do not evaluate zero-shot transfer. The SportsMOT and DanceTrack comparisons evaluate complete systems because the published trackers use different detectors and training scopes. The effects of individual ORAT components were evaluated separately on SportsMOT under a common configuration.

TDLP lists its SportsMOT training setting as Train, but the training splits for the complete system, including the reused detector and ReID model, could not be established. Its Training Scope is therefore marked N/R in the SportsMOT and DanceTrack comparison tables [49]. McByte++ uses a detector pretrained on the target dataset without additional training of the association stage. Because the detector’s exact training split is unclear, its Training Scope is also marked N/R for SportsMOT [71].

#### 4.5.1. Main Results on SportsMOT

SportsMOT shares challenges with pig datasets, including fast nonlinear motion, repeated occlusion, and similar appearances. We used it to evaluate ORAT under motion conditions different from those in pig videos. Test results for the base configuration were generated with epoch 11 and affinity threshold *τ* = −9.25 fixed after selection on SportsMOT validation.

The base ORAT configuration achieved HOTA of 82.6, DetA of 88.9, AssA of 76.7, and IDF1 of 87.7 on SportsMOT. HOTA, AssA, and IDF1 were lower than the optional ORAT (LT) extension’s 83.0, 77.4, and 88.1 in Table 10, but higher than those of the other comparison systems. Comparisons among ORAT configurations are analyzed separately in the SportsMOT common-baseline experiments.

#### 4.5.2. Component Experiments on SportsMOT

Table 11 uses the same seven configurations as the PigTrack component experiments. Recent memory, auxiliary matchability, and localization quality are added independently to the ID baseline. The base ORAT configuration combines ID, RM, AM, and LQ, whereas ORAT (LT) additionally includes LT. Validation HOTA is reported as the mean ± sample SD across training seeds 0–4. Tracker latency is reported as the mean ± sample SD across five runs of a representative seed model for each configuration.

Adding the recent-memory configuration increased mean validation HOTA from 82.476 for the ID baseline to 84.797, a gain of 2.322 points. This was the largest increase among the three independent additions to the ID baseline. The base configuration combining all four components achieved the highest validation HOTA at 84.932 ± 0.169. Adding the recent-memory configuration increased mean validation HOTA on both PigTrack and SportsMOT, but this comparison also includes differences in memory storage and cumulative identity-state updates.

On SportsMOT, mean validation HOTA was 84.932 for the base configuration and 84.866 for ORAT (LT). The base-minus-LT difference was +0.066 points, but its sequence-level paired bootstrap 95% CI, [−0.311, 0.461], included zero. The CI from the supplementary analysis using 13 source clusters also included zero at [−0.392, 0.541]. LT had higher latency at 2.377 ± 0.003 ms/frame, compared with 2.070 ± 0.003 ms/frame for the base configuration. The main results therefore consistently use the base configuration without LT, while LT is presented as an optional extension.

#### 4.5.3. Main Results on DanceTrack

DanceTrack contains nonlinear motion and frequent crossings of objects with similar appearances. It was used to evaluate detection and identity association under these conditions. The base configuration was trained separately on the DanceTrack training split, and epoch 19 and an affinity threshold of −9.5 were selected on its validation split. With the selected settings fixed, the model was trained on the combined training and validation splits to generate test results.

The base ORAT configuration achieved HOTA of 71.6, DetA of 85.6, AssA of 59.9, IDF1 of 75.5, and MOTA of 93.3 on DanceTrack. It had the highest DetA in Table 12, but lower HOTA and AssA than some comparison methods. Identity representations were generated within the detector, without external ReID, additional data, or external pose cues. Together with the SportsMOT results, this supports the ability of the proposed ORAT architecture to learn effective tracking on human datasets with different motion and occlusion conditions.

### 4.6. Qualitative Identity Continuity in Pig Videos

**Figure 5:**
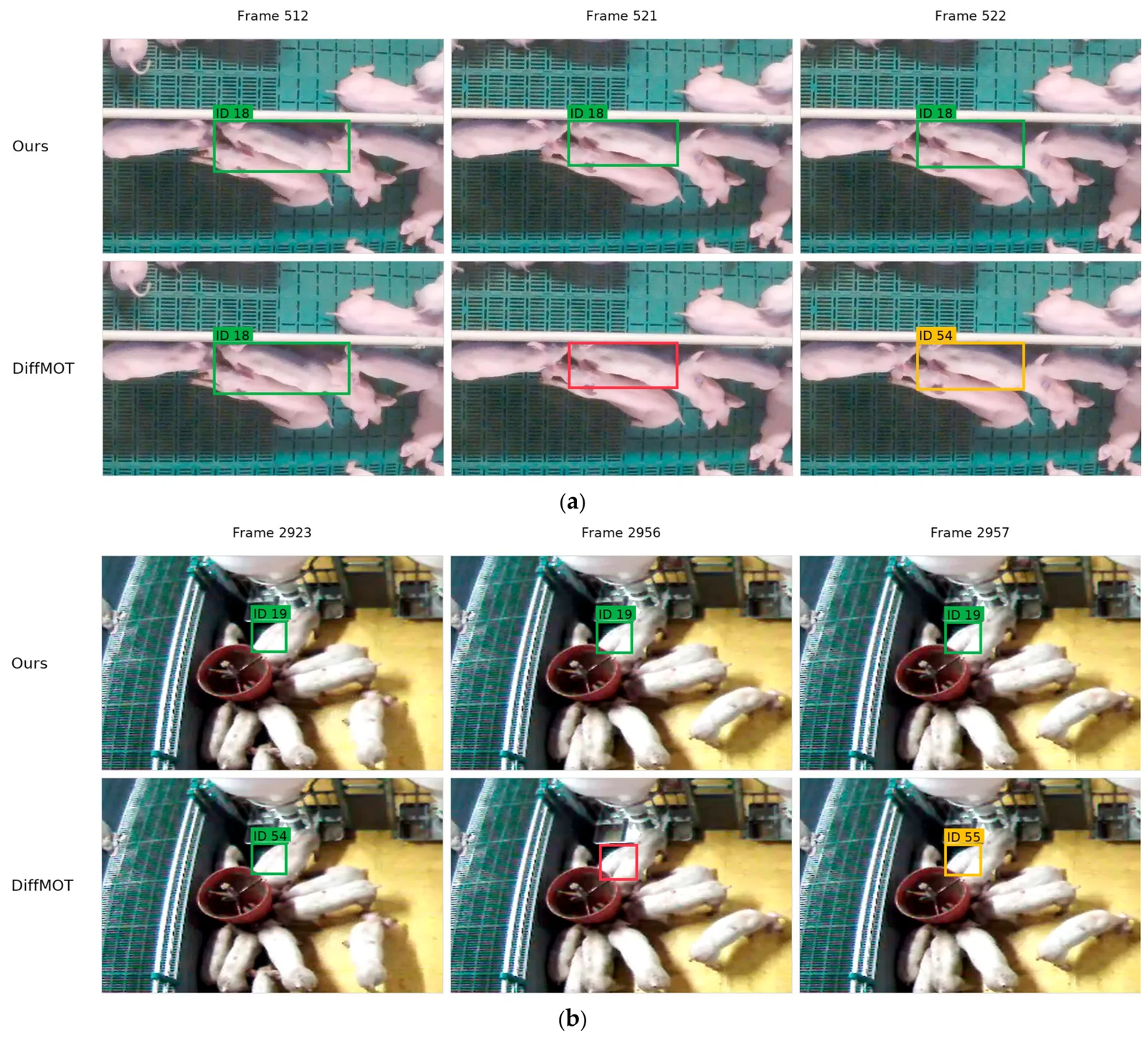
Identity continuity observed before and after temporary missed detections: (**a**) pigtrack0002 from PigTrack and (**b**) Video05 from Jochiwon23. Numbers are method-specific identity (ID) labels; the same number in different videos does not necessarily identify the same individual. Green indicates identity preservation, red a missed detection, and yellow a change to a different ID. ORAT, Observation- and Relation-based Affinity Tracker.

Figure 5 illustrates identity continuity before and after temporary missed detections. In PigTrack’s pigtrack0002 sequence, ORAT maintained ID 18 across frames 512, 521, and 522. DiffMOT assigned ID 18 before the missed detection and ID 54 afterward. In Jochiwon23’s Video05, ORAT maintained ID 19 across frames 2923, 2956, and 2957, whereas DiffMOT assigned ID 54 before the missed detection and ID 55 afterward. In both examples, ORAT preserved the existing identity when detections reappeared.

Figure 5 presents qualitative examples from the complete system, extracted separately from the quantitative comparison, and illustrates identity continuity when detections reappear after temporary misses.

## 5. Discussion

When posture, activity, social contact, and movement are aggregated by individual, ID changes and fragmented trajectories can cause confusion about which animal is being analyzed. Maintaining stable identity association is therefore directly relevant to the reliability of livestock analyses [5,6,7,8,76]. In the PigTrack comparison using identical D-FINE-X detections, ORAT achieved higher AssA and IDF1 than DiffMOT, but slightly lower DetA and MOTA [11,50]. The differences between the two methods can therefore be interpreted as arising primarily from identity association rather than improved detector outputs. The same pattern of metrics was observed on Jochiwon23 when models selected through training and validation were evaluated on separate outer-test blocks.

The controlled identity-representation experiments support this interpretation while distinguishing the comparison conditions. With the detector, bboxes, and dimensionality (256) held constant, the dedicated head output C achieved higher test HOTA than the pre-head query B, with the paired bootstrap CI entirely above zero. In the 512-dimensional comparison using the same detections, detector-side embeddings achieved higher HOTA and throughput than external OSNet [21]. External OSNet achieved higher standalone Rank-1, so retrieval metrics alone do not explain the association results. The results can instead be interpreted in terms of how the proposed method uses the extracted representations together with other tracking cues.

The recent-memory configuration increased mean validation HOTA across five seeds on both PigTrack and SportsMOT. This is consistent with the design of retaining individual observations and recomputing their relations with the current detection. However, the comparison also includes differences in memory storage and cumulative identity-state updates, requiring more detailed analysis of the underlying causes. In the PigTrack readout control, which separated these factors, the full relation configuration (R3), using three similarity statistics, occupancy, and the age of the most similar observation, achieved the highest validation HOTA among the four conditions.

The main results in Table 2, Table 3, Table 10 and Table 12 use the base configuration, while the long-term path is presented as the optional ORAT (LT) extension. The two configurations achieved comparable mean validation HOTA on PigTrack and SportsMOT, whereas LT achieved a slightly higher five-seed mean test HOTA on PigTrack and higher single-run test HOTA on SportsMOT and DanceTrack. On Jochiwon23, both configurations achieved identical accuracy in the outer evaluation, but LT exhibited lower throughput. The base configuration was selected based on its comparable validation performance and lower tracker latency. This selection was made independently of the test results.

In addition to testing on public PigTrack and outer evaluation on private Jochiwon23, we evaluated SportsMOT and DanceTrack to examine motion and occlusion conditions different from those in pig videos [11,22,23]. Models were trained independently on the two human tracking datasets, with settings selected on the respective validation splits. Published comparison methods differ in their detectors, training data, and auxiliary cues, so these results support applying the same association design to pig and human tracking after dataset-specific training. The base configuration generates identity representations within the detector and achieved a server full-pipeline throughput of 48.6 FPS, higher than the comparison configurations using separate appearance-extraction paths. On Jetson, it also achieved higher throughput than external ReID methods and higher HOTA than the faster FTO-SORT. These results show that tracking accuracy and throughput can be considered jointly without additional ReID inference, including on an edge device. Because detector sizes and device environments differ, accuracy and throughput are compared within each environment.

Limitations of the current design begin with candidate generation and memory updates. Conditional positive-pair recall was 100% for gates of 0.25–0.45 on PigTrack validation, but this measure is restricted to cases in which both a detection matched to the ground truth and an alive, eligible track exist. This result does not resolve the possibility that the gate may exclude pairs with large displacements in other scenes, or that detections or tracks themselves may be absent. Incorrectly accepted matches can also contaminate subsequent cumulative state and memory. The maximum missed-observation gap and the criterion for using LT relations are fixed in frames, so their actual temporal spans vary with video frame rates. The procedure also performs one-to-one assignment followed by affinity-threshold acceptance rather than directly solving an optimal partial-matching problem that includes the threshold in its objective.

Jochiwon23 is private data from a single farm and is not independent at the individual-animal level; inter-annotator agreement was not separately quantified. Future work should investigate motion-adaptive candidate ranges, memory updates resilient to incorrect matches, and evaluation across multiple farms, cameras, and growth stages [26,27].

## 6. Conclusions

We proposed the Observation- and Relation-based Affinity Tracker (ORAT) for online multi-pig tracking. ORAT generates box-aligned identity embeddings from the detector’s final queries. Each track maintains both cumulative state summarizing all accepted observations and bounded memory retaining individual accepted recent observations. For each current detection candidate, relations with stored observations are recomputed and combined with track and detection representations and geometry, confidence, and identity cues to produce an affinity score. Hungarian one-to-one assignment is followed by affinity-threshold acceptance, and only accepted matches update state and memory.

In the shared-detection comparison on the PigTrack test, the base ORAT configuration achieved a HOTA score of 88.1. On private Jochiwon23 videos, a higher AssA than DiffMOT was also observed in the two-direction outer evaluation separated from internal model selection. With separate training and model selection on SportsMOT and DanceTrack, the same architecture was applied to human tracking under motion and occlusion conditions different from those in pig videos. In the 5-seed component experiments on PigTrack and SportsMOT, adding recent memory increased mean validation HOTA. The PigTrack representation interface and readout controls supported the use of detector-side identity and per-candidate observation relations under their respective comparison conditions. LT is presented as an optional extension, separate from the main results. The detector-side interface requires neither separate bbox cropping nor a ReID forward pass, and the server full pipeline using D-FINE-X achieved 48.6 FPS. These results support ORAT as a design that improves identity association on pig datasets and can also be trained for human tracking.

## Figures and Tables

**Table 1:** Dataset sizes and evaluation roles. For Jochiwon23, 3/1/4 × 2 directions denotes the numbers of training/validation/outer-test sequences in each direction; both directional splits were applied to the same 8 sequences for evaluation. The SportsMOT frame and bbox counts are lower-bound totals reported by the original source, and 25 FPS for Jochiwon23 is the encoded frame rate of the processed videos. The Jochiwon23 splits are detailed in Section 4.1.2. Abbreviations: bbox, bounding box; FPS, frames per second; Seq., sequences; Obj., objects.

Dataset	Type/ Access	Seq. (Split)	Frames	Boxes	Obj./ Frame	Resolution; FPS	Role
PigTrack [11]	Pig/Public	80 (35/20/25)	26,487	517,961	19.6	1280 × 800; 5–15	Primary test
Jochiwon23	Pig/Private	8 (3/1/4 × 2 directions)	23,867	546,734	22.9	640 × 480; 25	Supplementary outer test
SportsMOT [22]	Person/Public	240 (45/45/150)	>150,000	>1,600,000	>10.7	1280 × 720; 25	Supplementary test
DanceTrack [23]	Person/Public	100 (40/25/35)	≈105,000	≈877,000	≈8.3	Various; 20	Supplementary test

**Table 2:** Local comparisons using shared detections and published system results on the PigTrack test split. The two comparison categories and † are defined in Section 4.1.4. The gray region contains published results; values for published baselines other than SIPTrack were taken from the PigTrack benchmark [11]. Citations in the Method column identify the original papers for the methods. ORAT denotes the base configuration, and ORAT (LT) denotes the optional long-term extension. Accuracy is the test result for each selected model, and E2E FPS is the mean of five measurements. Abbreviations: ORAT, Observation- and Relation-based Affinity Tracker; LT, long-term; HOTA, Higher Order Tracking Accuracy; DetA, detection accuracy; AssA, Association Accuracy; IDF1, Identification F1 Score; MOTA, Multiple Object Tracking Accuracy; E2E, end-to-end; FPS, frames per second. T denotes the target training split. E/I denote external/internal appearance paths; — denotes no learned appearance representation. N/R denotes a value not reported; in Training Scope, it indicates insufficient information to determine the training scope. Higher values are better (↑). Bold and underlined values indicate the best and second-best values, respectively, within the local-comparison and published-result sections separately.

Method	Training Scope	Appearance	HOTA ↑	DetA ↑	AssA ↑	IDF1 ↑	MOTA ↑	E2E FPS ↑
**Local comparisons (shared detections)**								
**Appearance source: —**								
ByteTrack ^†^ [40]	T	—	79.5	79.4	80.0	90.0	91.8	**50.6**
OC-SORT ^†^ [41]	T	—	85.2	86.7	84.0	90.0	93.4	49.7
FTO-SORT ^†^ [43]	T	—	83.8	82.1	85.9	93.1	91.7	45.6
**Appearance source: E**								
Hybrid-SORT ^†^ [63]	T	E	83.0	83.6	82.7	86.4	89.8	31.4
Deep-EIoU ^†^ [64]	T	E	84.3	85.9	82.9	89.5	93.4	32.4
Deep OC-SORT ^†^ [65]	T	E	85.7	86.8	84.7	90.5	93.4	34.8
BoT-SORT ^†^ [66]	T	E	86.5	86.4	86.8	92.5	**94.0**	33.9
DiffMOT ^†^ [50]	T	E	87.2	**87.1**	87.4	92.6	**94.0**	31.1
**Appearance source: I**								
ORAT (LT)	T	I	88.0	86.8	89.3	94.6	93.5	47.5
ORAT	T	I	**88.1**	86.8	**89.5**	**94.9**	93.5	48.6
**Published results**								
ByteTrack [40]	T	—	80.6	81.5	80.2	90.7	93.9	N/R
StrongSORT [67]	T	E	83.8	87.0	80.9	87.8	94.0	N/R
MOTIP [68]	T	I	83.5	84.6	82.6	89.7	91.2	N/R
OC-SORT [41]	T	—	86.8	88.4	85.4	91.4	95.3	N/R
Deep OC-SORT [65]	T	E	86.9	88.1	85.9	91.6	94.7	N/R
Improved Association [69]	T	E	87.3	**88.7**	86.1	92.1	95.9	N/R
MOTRv2 [70]	T	I	87.6	87.4	88.0	93.3	94.3	N/R
BoT-SORT [66]	T	E	87.9	88.5	87.4	93.5	**96.1**	N/R
SIPTrack [45]	N/R	I	**89.3**	88.5	**90.1**	**95.8**	95.6	N/R

**Table 3:** Shared-detection results from two-direction outer evaluation on Jochiwon23. The two-direction outer-evaluation (2D-Outer) split is defined in Section 4.1.2, and † is defined in Section 4.1.4. Results were aggregated with TrackEval across 8 outer-test sequences while preserving sequence boundaries, rather than by taking the arithmetic mean of directional HOTA scores. IDSW denotes the number of identity switches, and E2E FPS is the mean of five measurements. Abbreviations: ORAT, Observation- and Relation-based Affinity Tracker; LT, long-term; HOTA, Higher Order Tracking Accuracy; DetA, detection accuracy; AssA, Association Accuracy; IDF1, Identification F1 Score; MOTA, Multiple Object Tracking Accuracy; E2E, end-to-end; FPS, frames per second. E/I denote external/internal appearance paths; — denotes no learned appearance representation. Higher values are better (↑), except where lower values are indicated (↓). Bold and underlined values indicate the best and second-best values, respectively, within each comparison.

Method	Protocol	Appearance	HOTA ↑	DetA ↑	AssA ↑	IDF1 ↑	MOTA ↑	IDSW ↓	E2E FPS ↑
ByteTrack ^†^ [40]	2D-Outer	—	71.0	79.5	63.7	78.7	94.0	462	**82.3**
OC-SORT ^†^ [41]	2D-Outer	—	76.5	86.2	68.0	80.2	97.2	396	80.6
FTO-SORT ^†^ [43]	2D-Outer	—	70.9	73.5	68.6	80.8	84.0	**217**	71.0
Hybrid-SORT ^†^ [63]	2D-Outer	E	73.6	86.4	62.8	74.3	97.5	728	46.8
Deep-EIoU ^†^ [64]	2D-Outer	E	76.1	86.2	67.3	81.1	97.8	780	47.1
BoT-SORT ^†^ [66]	2D-Outer	E	77.6	85.7	70.4	83.0	97.6	266	49.7
Deep OC-SORT ^†^ [65]	2D-Outer	E	77.8	86.2	70.3	81.9	97.1	373	47.7
DiffMOT ^†^ [50]	2D-Outer	E	78.2	**86.7**	70.7	83.3	**97.8**	386	41.8
ORAT	2D-Outer	I	**79.1**	86.4	**72.6**	**84.9**	97.2	274	66.1
ORAT (LT)	2D-Outer	I	**79.1**	86.4	**72.6**	**84.9**	97.2	274	64.7

**Table 4:** Identity-representation comparison within the base architecture on PigTrack. B and C compare head paths with the detector, bboxes, and dimensionality (256) held constant; D and E compare external and internal 512-dimensional representations using the same detections. A tracker was trained separately for each condition with seed 0, and its checkpoint and affinity threshold were selected on validation. Abbreviations: ORAT, Observation- and Relation-based Affinity Tracker; bbox, bounding box; ID, identity; HOTA, Higher Order Tracking Accuracy; AssA, Association Accuracy; IDF1, Identification F1 Score; E2E, end-to-end; FPS, frames per second; SD, standard deviation; Dim., dimensionality; Val., validation. Higher values are better (↑). Bold and underlined values indicate the best and second-best values, respectively, within each comparison.

Representation	Dim.	Test Rank-1 (%) ↑	Val. HOTA ↑	Test HOTA ↑	Test AssA ↑	Test IDF1 ↑	E2E FPS ↑ (Mean ± SD)
A: Query from a detector trained without ID loss	256	15.351	94.310	87.702	88.479	94.148	**49.401 ± 0.120**
B: Pre-head query from the detector used in C	256	28.509	93.997	87.154	87.865	93.503	49.150 ± 0.162
C: detector-side ID head	256	23.246	94.398	88.054	**89.563**	**94.909**	49.307 ± 0.087
D: External OSNet-x1.0	512	**43.516**	94.226	87.289	87.990	93.606	33.967 ± 0.047
E: Detector-side ID head (ORAT)	512	22.857	**94.608**	**88.064**	89.468	94.884	48.615 ± 0.035

**Table 5:** PigTrack common-baseline component experiments. ID denotes detector-side identity, RM recent memory, AM the auxiliary matchability head used during training, LQ the localization-quality descriptor, and LT the optional long-term extension. Validation and test HOTA are reported as the mean ± sample SD across fixed training seeds 0–4. Abbreviations: ORAT, Observation- and Relation-based Affinity Tracker; HOTA, Higher Order Tracking Accuracy; SD, standard deviation; Val., validation. A check mark indicates that the component is enabled; — indicates that it is not enabled. Higher values are better (↑), except where lower values are indicated (↓). Bold and underlined values indicate the best and second-best values, respectively, within each comparison.

Configuration	ID	RM	AM	LQ	LT	Val. HOTA ↑ (5 Seeds)	Test HOTA ↑ (5 Seeds)	Tracker Latency ↓ (ms/Frame, 5 Runs)
State–geometry baseline	—	—	—	—	—	94.117 ± 0.101	87.109 ± 0.199	**2.775 ± 0.009**
ID baseline	✓	—	—	—	—	94.148 ± 0.104	87.922 ± 0.116	2.894 ± 0.008
Recent memory	✓	✓	—	—	—	94.599 ± 0.053	**88.147 ± 0.086**	2.780 ± 0.005
Matchability	✓	—	✓	—	—	94.150 ± 0.095	87.680 ± 0.503	2.928 ± 0.007
Localization quality	✓	—	—	✓	—	94.070 ± 0.089	87.999 ± 0.174	2.896 ± 0.006
ORAT	✓	✓	✓	✓	—	**94.622 ± 0.024**	88.052 ± 0.031	2.882 ± 0.007
ORAT (LT)	✓	✓	✓	✓	✓	94.606 ± 0.024	88.106 ± 0.136	3.221 ± 0.008

**Table 6:** PigTrack recent-observation readout control. All four conditions retain the memory-bank and running-mean state storage and update rules while changing the relation inputs to Pair-Bias. R0 uses no readout, R1 uses three similarity cues, R2 uses occupancy and best-observation age, and R3 uses all five cues. Accuracy is reported for models trained with seed 0 and selected on validation; E2E FPS is the mean ± sample SD across 5 live-pipeline runs. R3 has the same configuration as the base ORAT used for the main results. Abbreviations: ORAT, Observation- and Relation-based Affinity Tracker; HOTA, Higher Order Tracking Accuracy; AssA, Association Accuracy; IDF1, Identification F1 Score; E2E, end-to-end; FPS, frames per second; SD, standard deviation; Val., validation. Higher values are better (↑). Bold and underlined values indicate the best and second-best values, respectively, within each comparison.

Readout	Relation Cues Used	Val. HOTA ↑	Test HOTA ↑	Test AssA ↑	Test IDF1 ↑	E2E FPS ↑ (Mean ± SD)
R0	None (bank and running mean retained)	94.155	86.923	87.264	93.068	**50.429 ± 0.056**
R1	Max/top-k mean/similarity gap	94.575	**88.370**	**90.091**	**95.527**	48.662 ± 0.101
R2	Occupancy/best-observation age	94.163	87.340	88.068	93.827	48.624 ± 0.104
R3	All 5 cues	**94.608**	88.064	89.468	94.884	48.615 ± 0.035

**Table 7:** Geometric-gate sensitivity of the same base model on PigTrack validation. Gate denotes the upper bound on center distance normalized by the image diagonal. Conditional recall is restricted to positive pairs with both a corresponding detection and an alive, eligible track. Candidate counts are reported as the mean per frame and the 95th percentile (p95); tracker latency is the mean ± sample SD across 5 cache-based closed-loop measurements. Model training and the affinity threshold were held fixed. Abbreviations: HOTA, Higher Order Tracking Accuracy; SD, standard deviation; Val., validation. Higher values are better (↑).

Gate	Val. HOTA ↑	Conditional Recall (%) ↑	Mean Candidates/Frame	Candidate p95	Tracker Latency (ms/Frame, Mean ± SD)
0.25	94.609	100.000	183.6	349	2.657 ± 0.005
0.30	94.608	100.000	227.3	416	2.664 ± 0.006
0.35 (default)	94.609	100.000	268.1	481	2.658 ± 0.008
0.40	94.609	100.000	303.8	538	2.666 ± 0.010
0.45	94.608	100.000	334.0	590	2.665 ± 0.007

**Table 8:** Model complexity and processing cost of base ORAT in the PigTrack server profile. Parameter counts include registered unique parameters in the deployed objects. Neural GFLOPs sum MAC × 2 for convolutions and matrix multiplications and the specified elementwise approximations. The association value is a frame-weighted mean based on the actual track/detection shapes observed across 8939 frames from 25 test sequences. Latencies are independent measurements at the boundaries specified for each row, and the full pipeline is measured directly over the complete path. Tracker and full-pipeline latencies are the mean ± sample SD across 5 runs of the base configuration. Abbreviations: ORAT, Observation- and Relation-based Affinity Tracker; GFLOPs, billions of floating-point operations; MAC, multiply–accumulate operation; SD, standard deviation; ID, identity; Params, parameters; M, million. Lower values are preferred (↓).

Module/Scope	Params (M) ↓	Neural GFLOPs/Frame ↓	Latency (ms/Frame) ↓	Measurement Boundary
Detector path	61.541	500.409	16.324	Prepared input → detections (excluding ID head)
ID head	0.198	0.119	0.050	[1,300,256] queries → [1,300,512] ID embeddings
Association tracker	0.257	0.016	2.788 ± 0.008	Cached detector outputs → track states
Added modules	0.455	0.135	—	Combined complexity of ID head + tracker
Full pipeline	61.996	500.545	20.570 ± 0.015	Live decode → synchronized tracks

**Table 9:** Local PigTrack comparison on Jetson Orin Nano Super using D-FINE-N at 640 × 640 and TensorRT FP16. TensorRT FP16 is also used for external ReID models. ORAT denotes the base configuration, and ORAT (LT) denotes the optional long-term extension. Abbreviations: ORAT, Observation- and Relation-based Affinity Tracker; LT, long-term; ReID, re-identification; FP16, 16-bit floating-point precision; HOTA, Higher Order Tracking Accuracy; DetA, detection accuracy; AssA, Association Accuracy; IDF1, Identification F1 Score; MOTA, Multiple Object Tracking Accuracy; E2E, end-to-end; FPS, frames per second. E/I denote external/internal appearance paths; — denotes no learned appearance representation. Higher values are better (↑), except where lower values are indicated (↓). Bold and underlined values indicate the best and second-best values, respectively, within each comparison.

Method	Appearance	HOTA ↑	DetA ↑	AssA ↑	IDF1 ↑	MOTA ↑	E2E FPS ↑	E2E Latency ↓ (ms/Frame)
ByteTrack	—	69.0	68.1	70.4	79.7	80.6	**27.8**	**35.9**
OC-SORT	—	70.9	70.2	72.0	78.1	78.9	22.1	45.3
Deep-EIoU	E	70.6	72.5	69.1	77.4	82.3	4.9	203.1
Deep OC-SORT	E	71.1	70.2	72.4	78.1	78.9	8.7	114.5
Hybrid-SORT	E	71.2	70.9	72.0	76.8	78.2	5.0	200.8
BoT-SORT	E	74.0	72.9	**75.6**	81.8	83.0	9.2	108.4
DiffMOT	E	**74.2**	73.5	75.4	**81.9**	83.1	5.9	168.6
FTO-SORT	—	70.0	66.0	74.6	79.2	76.2	18.4	54.4
ORAT	I	74.1	**74.0**	74.7	81.1	83.2	16.3	61.3
ORAT (LT)	I	74.0	73.8	74.6	81.2	**83.3**	14.7	67.8

**Table 10:** Online tracker results on the SportsMOT test split. Training Scope, Appearance, and Extra are defined in Section 4.1.4. Each row reflects the complete configuration of the corresponding system. ORAT denotes the base configuration, and ORAT (LT) denotes the optional long-term extension; both report test results for models trained and selected on SportsMOT. ByteTrack and OC-SORT values were taken from the SportsMOT benchmark [22], and the Method column cites the original papers for the methods. Abbreviations: ORAT, Observation- and Relation-based Affinity Tracker; LT, long-term; HOTA, Higher Order Tracking Accuracy; DetA, detection accuracy; AssA, Association Accuracy; IDF1, Identification F1 Score; MOTA, Multiple Object Tracking Accuracy. T/V denote training/validation splits; T + V indicates optimization on both splits. N/R denotes a value not reported; in Training Scope, it indicates insufficient information to determine the training scope. E/I denote external/internal appearance paths; — denotes no learned appearance representation. A check mark in Extra indicates additional data or task-specific supervision. Higher values are better (↑). Bold and underlined values indicate the best and second-best values, respectively, within each comparison.

Method	Training Scope	Appearance	Extra	HOTA ↑	DetA ↑	AssA ↑	IDF1 ↑	MOTA ↑
**Appearance source: —**								
ByteTrack [40]	T + V	—	—	64.1	78.5	52.3	71.4	95.9
OC-SORT [41]	T + V	—	—	73.7	88.5	61.5	74.0	96.5
**Appearance source: E**								
PuTR [57]	T	E	—	74.3	87.2	63.4	75.7	95.2
DiffMOT [50]	T + V	E	—	76.2	**89.3**	65.1	76.1	**97.1**
Deep-EIoU [64]	T + V	E	—	77.2	88.2	67.7	79.8	96.3
McByte++ Online [71]	N/R	E	✓	79.9	N/R	N/R	83.6	96.9
CAMELTrack [72]	T + V	E	—	80.4	88.8	72.8	84.8	96.3
NOOUGAT-Online [73]	N/R	E	—	81.0	N/R	74.3	85.3	96.0
HAT-OC-SORT-ReID [19]	N/R	E	—	81.2	87.2	75.6	N/R	N/R
TDLP [49]	N/R	E	✓	81.9	88.0	76.3	87.5	95.8
MeMoSORT [74]	N/R	E	✓	82.1	**89.3**	75.6	86.4	97.0
HAT-OC-SORT-ReID [19]	T + V	E	—	82.4	**89.3**	76.1	N/R	N/R
**Appearance source: I**								
MeMOTR [55]	T	I	—	70.0	83.1	59.1	71.4	91.5
TBDQ-Net [51]	T	I	—	72.1	82.8	62.8	72.9	89.5
MOTIP [68]	T	I	—	72.6	83.5	63.2	77.1	92.4
FDTA [39]	T	I	✓	74.2	84.1	65.5	78.5	93.0
TBDQ-Net [51]	T + V	I	—	75.5	85.9	66.5	76.1	92.9
ORAT	T + V	I	—	82.6	88.9	76.7	87.7	96.1
ORAT (LT)	T + V	I	—	**83.0**	89.0	**77.4**	**88.1**	96.1

**Table 11:** SportsMOT common-baseline component experiments. The configurations are the same as in Table 5. ID denotes detector-side identity, RM recent memory, AM the auxiliary matchability head used during training, LQ the localization-quality descriptor, and LT the optional long-term extension. Validation HOTA is reported as the mean ± sample SD across training seeds 0–4. Latency is the mean runtime for a representative seed. Abbreviations: ORAT, Observation- and Relation-based Affinity Tracker; HOTA, Higher Order Tracking Accuracy; SD, standard deviation; Val., validation. A check mark indicates that the component is enabled; — indicates that it is not enabled. Higher values are better (↑), except where lower values are indicated (↓). Bold and underlined values indicate the best and second-best values, respectively, within each comparison.

Configuration	ID	RM	AM	LQ	LT	Val. HOTA ↑ (5 Seeds)	Tracker Latency ↓ (ms/Frame, 5 Runs)
State–geometry baseline	—	—	—	—	—	78.028 ± 0.338	**1.715 ± 0.006**
ID baseline	✓	—	—	—	—	82.476 ± 0.312	1.795 ± 0.003
Recent memory	✓	✓	—	—	—	84.797 ± 0.052	2.028 ± 0.003
Matchability	✓	—	✓	—	—	82.697 ± 0.322	1.825 ± 0.003
Localization quality	✓	—	—	✓	—	82.634 ± 0.139	1.805 ± 0.003
ORAT	✓	✓	✓	✓	—	**84.932 ± 0.169**	2.070 ± 0.003
ORAT (LT)	✓	✓	✓	✓	✓	84.866 ± 0.107	2.377 ± 0.003

**Table 12:** Online tracker results on the DanceTrack test split. Training Scope, Appearance, and Extra are defined in Section 4.1.4. Each result reflects the complete configuration of the corresponding system. ORAT reports results for the base configuration independently trained and selected on DanceTrack, and ORAT (LT) reports results for the optional extension. ByteTrack values were taken from the DanceTrack benchmark [23], and the Method column cites the original papers for the methods. Abbreviations: ORAT, Observation- and Relation-based Affinity Tracker; LT, long-term; HOTA, Higher Order Tracking Accuracy; DetA, detection accuracy; AssA, Association Accuracy; IDF1, Identification F1 Score; MOTA, Multiple Object Tracking Accuracy. T/V denote training/validation splits; T + V indicates optimization on both splits. N/R denotes a value not reported; in Training Scope, it indicates insufficient information to determine the training scope. E/I denote external/internal appearance paths; — denotes no learned appearance representation. A check mark in Extra indicates additional data or task-specific supervision. Higher values are better (↑). Bold and underlined values indicate the best and second-best values, respectively, within each comparison.

Method	Training Scope	Appearance	Extra	HOTA ↑	DetA ↑	AssA ↑	IDF1 ↑	MOTA ↑
**Appearance source: —**								
ByteTrack [40]	T	—	—	47.7	71.0	32.1	53.9	89.6
OC-SORT [41]	T	—	—	55.1	80.3	38.3	54.6	92.0
C-TWiX [48]	T	—	—	62.1	81.8	47.2	63.6	91.4
**Appearance source: E**								
PuTR [57]	T	E	—	60.6	82.6	44.6	61.7	92.3
Deep OC-SORT [65]	N/R	E	—	61.3	82.2	45.8	61.5	92.3
DiffMOT [50]	N/R	E	—	62.3	82.5	47.2	63.0	92.8
Hybrid-SORT-ReID [63]	N/R	E	✓	65.7	N/R	N/R	67.4	91.8
NOOUGAT-Online [73]	N/R	E	—	65.9	N/R	54.9	70.6	88.9
CAMELTrack [72]	T + V	E	—	66.1	81.1	54.0	71.1	91.4
TrackTrack [75]	N/R	E	—	66.5	N/R	52.9	67.8	**93.6**
HAT-Hybrid-SORT-ReID [19]	N/R	E	—	66.9	81.5	55.0	71.3	90.5
MeMoSORT [74]	N/R	E	✓	67.9	85.0	54.3	68.0	93.4
TDLP [49]	N/R	E	✓	70.1	82.6	59.6	75.8	91.8
**Appearance source: I**								
MeMOTR [55]	T	I	—	68.5	80.5	58.4	71.2	89.9
TBDQ-Net [51]	T	I	—	69.4	80.8	59.8	73.4	89.5
MOTIP [68]	T	I	—	69.6	80.4	60.4	74.7	90.6
FDTA [39]	T	I	✓	71.7	81.0	63.5	77.2	91.3
MOTIP [68]	T	I	✓	72.0	81.8	63.5	76.8	91.9
FDTA [39]	T + V	I	✓	**74.4**	82.7	**67.0**	**80.0**	92.2
ORAT	T + V	I	—	71.6	**85.6**	59.9	75.5	93.3
ORAT (LT)	T + V	I	—	72.0	85.2	60.9	76.1	93.3

## Data Availability

The public datasets used in this study are available from the sources cited in the manuscript. A limited selection of representative Jochiwon23 frames, ROI mask examples, and qualitative results is presented with permission. The complete raw and processed videos, box annotations, within-sequence identity labels, ROI masks, and derived detector and tracker caches are unavailable because the data owner has not authorized their release. At the time of submission, there are no plans to distribute the complete Jochiwon23 dataset publicly or through restricted access. The short public sequence distributed with FTO-SORT is separate material and does not provide access to the eight sequences used in this study.
